# Iron quantification in basal ganglia: quantitative susceptibility mapping as a potential biomarker for Alzheimer’s disease – a systematic review and meta-analysis

**DOI:** 10.3389/fnins.2024.1338891

**Published:** 2024-02-26

**Authors:** Sadegh Ghaderi, Sana Mohammadi, Nahid Jashire Nezhad, Shaghayegh Karami, Fatemeh Sayehmiri

**Affiliations:** ^1^Department of Neuroscience and Addiction Studies, School of Advanced Technologies in Medicine, Tehran University of Medical Sciences, Tehran, Iran; ^2^Department of Medical Sciences, School of Medicine, Iran University of Medical Sciences, Tehran, Iran; ^3^The Persian Gulf Tropical Medicine Research Center, The Persian Gulf Biomedical Sciences Research Institute, Bushehr University of Medical Sciences, Bushehr, Iran; ^4^School of Medicine, Tehran University of Medical Sciences, Tehran, Iran; ^5^Skull Base Research Center, Loghman Hakim Hospital, Shahid Beheshti University of Medical Science, Tehran, Iran

**Keywords:** Alzheimer’s disease, basal ganglia, iron, MRI, QSM

## Abstract

**Introduction:**

Alzheimer’s disease (AD), characterized by distinctive pathologies such as amyloid-β plaques and tau tangles, also involves deregulation of iron homeostasis, which may accelerate neurodegeneration. This meta-analysis evaluated the use of quantitative susceptibility mapping (QSM) to detect iron accumulation in the deep gray matter (DGM) of the basal ganglia in AD, contributing to a better understanding of AD progression, and potentially leading to new diagnostic and therapeutic approaches.

**Methods:**

Using the Preferred Reporting Items for Systematic Reviews and Meta-Analyses (PRISMA) guidelines, we systematically searched the PubMed, Scopus, Web of Sciences, and Google Scholar databases up to October 2023 for studies employing QSM in AD research. Eligibility criteria were based on the PECO framework, and we included studies assessing alterations in magnetic susceptibility indicative of iron accumulation in the DGM of patients with AD. After initial screening and quality assessment using the Newcastle-Ottawa Scale, a meta-analysis was conducted to compare iron levels between patients with AD and healthy controls (HCs) using a random-effects model.

**Results:**

The meta-analysis included nine studies comprising 267 patients with AD and 272 HCs. There were significantly higher QSM values, indicating greater iron deposition, in the putamen (standardized mean difference (SMD) = 1.23; 95% CI: 0.62 to 1.84; *p* = 0.00), globus pallidus (SMD = 0.79; 95% CI: 0.07 to 1.52; *p* = 0.03), and caudate nucleus (SMD = 0.72; 95% CI: 0.39 to 1.06; *p* = 0.00) of AD patients compared to HCs. However, no significant differences were found in the thalamus (SMD = 1.00; 95% CI: −0.42 to 2.43; *p* = 0.17). The sensitivity analysis indicated that no single study impacted the overall results. Age was identified as a major contributor to heterogeneity across all basal ganglia nuclei in subgroup analysis. Older age (>69 years) and lower male percentage (≤30%) were associated with greater putamen iron increase in patients with AD.

**Conclusion:**

The study suggests that excessive iron deposition is linked to the basal ganglia in AD, especially the putamen. The study underscores the complex nature of AD pathology and the accumulation of iron, influenced by age, sex, and regional differences, necessitating further research for a comprehensive understanding.

## 1 Introduction

Alzheimer’s disease (AD) is a progressive brain disorder that leads to memory loss, cognitive function decline, and behavioral alterations (Ávila-Villanueva et al., 2022). It is not a normal part of aging and is one of the leading causes of death in the United States, ranking seventh overall (Doblhammer et al., 2022). AD is the most common type of dementia, accounting for 60–80% of cases (DeTure and Dickson, 2019; Alzheimer’s Association, 2023). Less than half of these cases are pure AD, with the majority mixed with dementia (DeTure and Dickson, 2019). The World Health Organization (WHO) predicts that the global number of people with dementia is currently around 55 million and is projected to increase to approximately 78 million by 2030 and 139 million by 2050 (World Health Organization, 2023). By 2050, it is projected that 71% of individuals with dementia will reside in low-or middle-income countries compared to 58% in 2010 (Prince et al., 2013). Estimates indicate that there will be a 117% increase in the prevalence of dementia across all age groups from 2019 to 2050, highlighting the increasing difficulty of the situation (Nichols et al., 2022).

At the core of AD pathology is progressive neuronal loss within specific cerebral domains, which is characterized by the accumulation of extracellular amyloid-β plaques and intracellular neurofibrillary tangles composed of hyperphosphorylated tau proteins. These pathological hallmarks are implicated in neuroinflammation, oxidative stress, disrupted synaptic communication, and the consequent neuronal death (Rajmohan and Reddy, 2017; Kinney et al., 2018; Goel et al., 2022). Another critical but less highlighted aspect of AD pathology is deregulation of iron homeostasis. Iron, an essential element for brain function (Ravanfar et al., 2021; Ghaderi et al., 2023), can be neurotoxic in excess (Salvador et al., 2010). Notably, patients with AD exhibit pronounced iron accumulation in brain regions, such as the cortex and basal ganglia (Ward et al., 2014; Liu et al., 2018), which is thought to exacerbate neurodegeneration (Ndayisaba et al., 2019; Wang et al., 2020).

Recent advances in post-processing neuroimaging methods have provided a novel tool for assessing brain iron concentration (Ghaderi et al., 2023). The relevance of iron accumulation in AD pathology warrants a sophisticated approach to its quantification, which is now feasible with advancements in magnetic resonance imaging (MRI) technologies (Ghaderi and Mohammadi, 2023; Mohammadi and Ghaderi, 2023), particularly quantitative susceptibility mapping (QSM) (Ghaderi et al., 2023). QSM provides quantitative measurements of tissue iron content, overcoming limitations of other MRI techniques such as R2* and phase imaging for quantifying iron (Liu et al., 2015; Ravanfar et al., 2021; Ghaderi et al., 2023). QSM allows for the precise measurement of magnetic susceptibility (χ), a property that reflects the relative ability of a tissue to alter its magnetic field (Wang et al., 2017). As iron is highly susceptible, QSM can be used to detect and quantify iron deposition in the brain (Cogswell et al., 2021; Ravanfar et al., 2021).

The basal ganglia, comprising deep gray matter (DGM) structures such as the putamen (PUT), globus pallidus (GP), caudate nucleus (CN), and thalamus, are integral to motor and cognitive processes (Leisman et al., 2014), and iron-rich environments make them susceptible to iron overload in AD (Daglas and Adlard, 2018; Liu et al., 2018). Iron dysregulation in these nuclei could feasibly influence the motor-related symptoms and cognitive deficits frequently observed in patients with AD (Ndayisaba et al., 2019; Uchida et al., 2022). For instance, motor impairments, although not as prominent as cognitive decline in AD, are a feature of the disease, and the basal ganglia’s role in motor function suggests that iron deposition could be a contributing factor (Schipper, 2012; Daugherty and Raz, 2015). Likewise, the involvement of the basal ganglia in cognitive functions such as executive control and procedural memory implies that iron accumulation may underpin some of the cognitive deficits in AD (Leisman et al., 2014; Sokolovič et al., 2023).

In this meta-analysis, we aimed to systematically evaluate the ability of QSM to detect iron accumulation in the DGM nuclei of patients with AD compared with healthy controls (HCs). We will synthesize findings from multiple studies to provide a comprehensive assessment of the role of QSM in iron dyshomeostasis in the basal ganglia of AD patients. By understanding the relationship between iron accumulation and AD pathology, we can gain valuable insights into the mechanisms underlying AD development and progression, potentially leading to new diagnostic and therapeutic strategies for this devastating disease.

## 2 Methods

### 2.1 Search strategy

The research adhered to the guidelines of Preferred Reporting Items for Systematic Reviews and Meta-Analyses (PRISMA) (Page et al., 2021). Systematic searches were conducted in databases, including PubMed, Scopus, Web of Sciences, and Google Scholar, to locate pertinent studies published up to October 2023. The search terms focused on QSM and AD, encompassing terms such as “Alzheimer’s disease,” “quantitative susceptibility mapping” OR “QSM,” and some brain regions such as “basal ganglia,” “striatum,” “caudate nucleus,” “putamen,” “globus pallidus,” “substantia nigra pars reticulata,” “subthalamic nucleus,” “thalamus,” “red nucleus,” “substantia nigra pars compacta,” and “substantia nigra.” The search strategy was tailored for each database (Supplementary Table S1). Gray literature, including dissertations, preprints, and conference papers, were explored using ProQuest and Scopus. The included articles were subjected to additional analyses using forward and backward citation tracking.

### 2.2 Eligibility criteria

The study formulated its inclusion and exclusion criteria and research questions by employing the Population, Exposure, Comparison, and Outcome (PECO) framework. All studies evaluating alterations in magnetic susceptibility in the DGM (basal ganglia nuclei) (Outcome) through QSM (Exposure) in patients with AD (Population) and Controls (Comparison) were considered eligible for inclusion, with no language restrictions. Exclusion criteria comprised Books, letters, notes, conference abstracts, editorials, surveys, case reports, series, animal studies, non-original research, and reviews were excluded. Additionally, studies utilizing alternative quantitative MRI methods, such as R2*, and lacking specific mention of QSM values in the basal ganglia nuclei were also excluded.

### 2.3 Screening and study selection

One author (SM) conducted the screening of titles and abstracts to identify studies that used QSM in AD to quantify iron in the nuclei of the DGM, specifically including the PUT, GP, CN, and thalamus. The selection process was independently conducted by S.G. and S.M. and any discrepancies were resolved through discussion. Two independent reviewers (S.G. and S.M.) screened the full texts to identify studies that fulfilled the eligibility criteria. The reference lists of the eligible studies were manually scrutinized for relevant publications through citation searches.

### 2.4 Data extraction and quality assessment

Three authors (SM, NN, and SK.) collected the data extracted from each study. The main data extraction was organized into several subdivisions that met the eligibility requirements, with a focus on the study’s characteristics, such as the first author’s name, publication year, country of the first author’s affiliation, field strengths, coil channels, subjects (patients and HCs), and basal ganglia nuclei QSM values. Two authors independently assessed the potential for bias using the Newcastle-Ottawa Scale (NOS) (Wells et al., 2000; Modesti et al., 2016). Discrepancies were resolved through discussions. The NOS encompasses three domains: selection (scoring ranging from 0 to 5 for cross-sectional studies and 0 to 4 for cohort and case–control studies), comparability (scoring from 0 to 2), and outcome (scoring from 0 to 3). Depending on the cumulative scores attained, the studies were classified into three distinct groups: those with a very high risk of bias (0 to 3 points), high risk of bias (4 to 6 points), and low risk of bias (7 to 10 points) (Herzog et al., 2013; Parasuaraman et al., 2023).

### 2.5 Meta-analysis

This meta-analysis aimed to compare the iron QSM values in patients with AD and HCs in different regions of the basal ganglia, including the PUT, GP, CN, and thalamus. Analysis was conducted using Stata version 17 (StataCorp, College Station, TX, United States). After data extraction, a meta-analysis was performed to determine whether there was sufficient data for a specific region. The standardized mean difference (SMD) between the patient and control groups was used to analyze iron levels. The cutoff values set by Cohen’s d were used to interpret small, medium, and large effect sizes (0.2, 0.5, and 0.8, respectively) (Cohen, 1988). A random-effects model was used for all the analyses. Heterogeneity was assessed using I^2^ statistics, and values greater than 50% were considered to indicate moderate-to-high heterogeneity. Subgroup analyses were performed to examine the origins of heterogeneity among the studies, focusing on variables such as age, sex, region, and risk of bias. Additionally, sensitivity analysis was performed to evaluate the impact of excluding each study on the overall outcomes. Publication bias was assessed by visual inspection of funnel plots and quantitative examination using the Egger’s regression test (Egger et al., 1997).

## 3 Results

### 3.1 Overview of results

**Figure 1:**
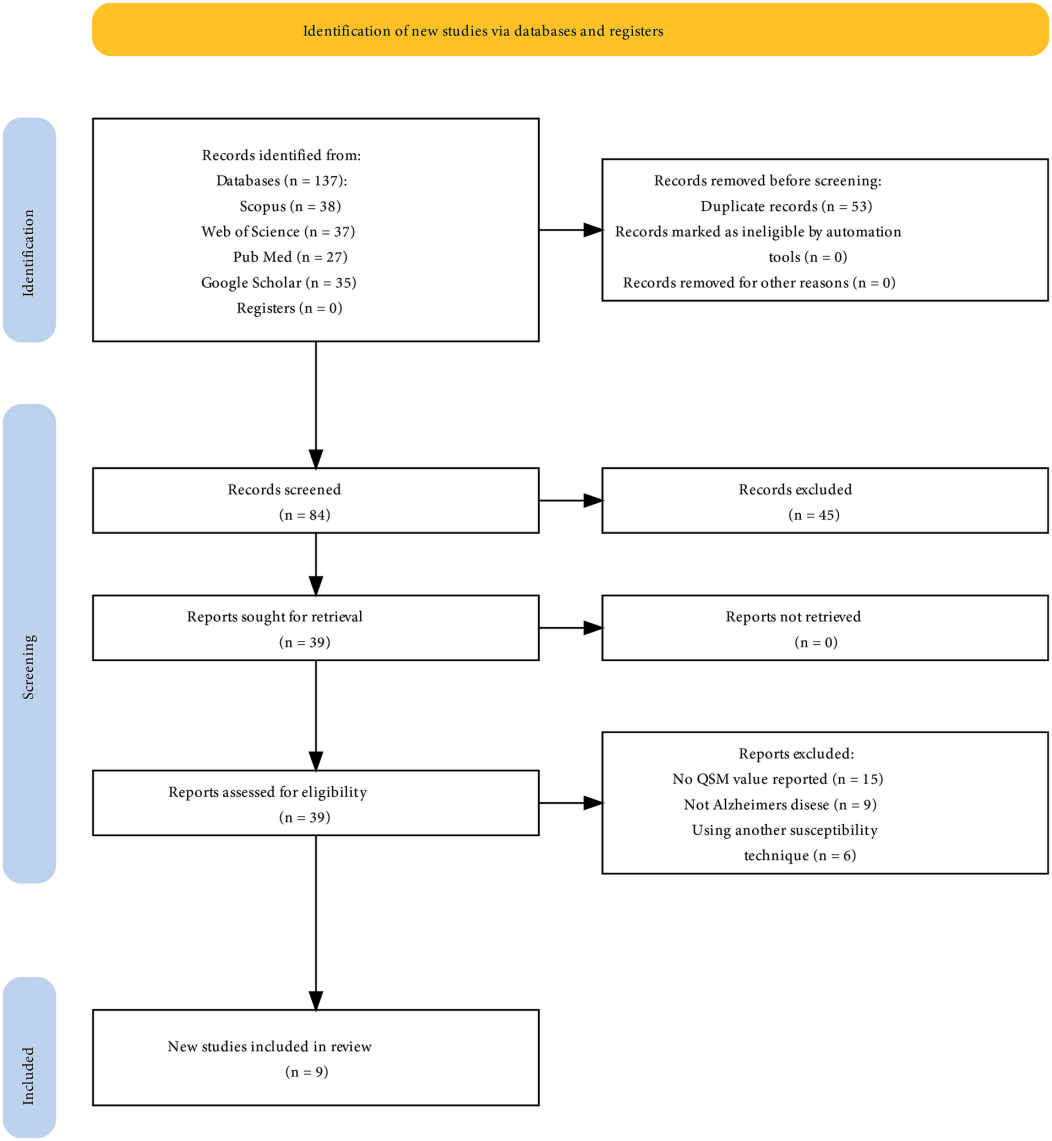
PRISMA flow diagram for systematic review.

A meta-analysis was conducted using nine studies (Figure 1) involving 267 patients with AD and 272 HCs. The characteristics and QSM values from these studies are presented in Table 1. All the studies utilized a magnetic field strength of 3 T. The QSM values have been reported for different basal ganglia, such as the PUT, GP, CN, and thalamus.

**Table 1:** QSM values for basal ganglia nuclei.

References	Year	Country	FS	Coil	Number of patients	Mean age (patients)	Gender (male)	Number of controls	PUT (P) QSM value ± SD	PUT (C) QSM value ± SD	GP (P) QSM value ± SD	GP (C) QSM value ± SD	CN (P) QSM value ± SD	CN (C) QSM value ± SD	Th (P) QSM value ± SD	Th (C) QSM value ± SD
Huang et al. (2023)	2023	China	3	NR	43	62.63	13	27	64.5 ± 13.05	56.5 ± 13.9	102 ± 13.8	97 ± 9.55	52.5 ± 8.8	45.5 ± 8.5	NR	NR
Yamaguchi et al. (2023)	2023	Japan	3	NR	37	75.7	20	37	45 ± 14.9	37.6 ± 14.6	NR	NR	NR	NR	NR	NR
Sharma et al. (2023)	2023	Canada	3	32	14	68.8	5	83	88 ± 30	61 ± 50	133 ± 20	107 ± 70	57 ± 30	46 ± 30	1 ± 10	-1 ± 10
Liu et al. (2021)	2021	China	3	8	59	71.12	21	22	NR	NR	NR	NR	NR	NR	220.36 ± 40.34	193.62 ± 40.99
Li et al. (2020)	2020	China	3	32	22	71.5	9	25	89 ± 24	31 ± 24	84 ± 15	33 ± 24	46 ± 11	23 ± 19	5 ± 13	-4 ± 11
Tiepolt et al. (2020)	2020	Germany	3	NR	16	69	4	11	49 ± 33	2 ± 31	NR	NR	58 ± 39	51 ± 39	NR	NR
Du et al. (2018)	2018	China	3	8	30	68.3	10	30	99.18 ± 31.35	81.17 ± 31.35	168.61 ± 26.01	168.89 ± 17.1	58.62 ± 18.17	50.64 ± 10.69	24.6 ± 12.11	32.38 ± 30.46
Kim et al. (2017)	2017	South Korea	3	8	19	69.79	2	19	3.77 ± 2.85	−3.23 ± 2.04	44.09 ± 4.56	38.04 ± 4.67	NR	NR	−19.91 ± 2.08	−30.15 ± 2.12
Moon et al. (2016)	2016	South Korea	3	NR	27	78.63	4	18	98.9 ± 33.63	58.48 ± 24.01	138.63 ± 36.24	126.78 ± 32.44	83.44 ± 22.44	63.96 ± 16.38	41.76 ± 21.19	36.71 ± 18.95

Abbreviations: QSM Value → parts per billion (ppb)/P: patient and C: control/CN, caudate nucleus; FS, field strength; GP, globus pallidus; HC, healthy control; NR, not reported; PUT, putamen; QSM, quantitative susceptibility mapping.

**Figure 2:**
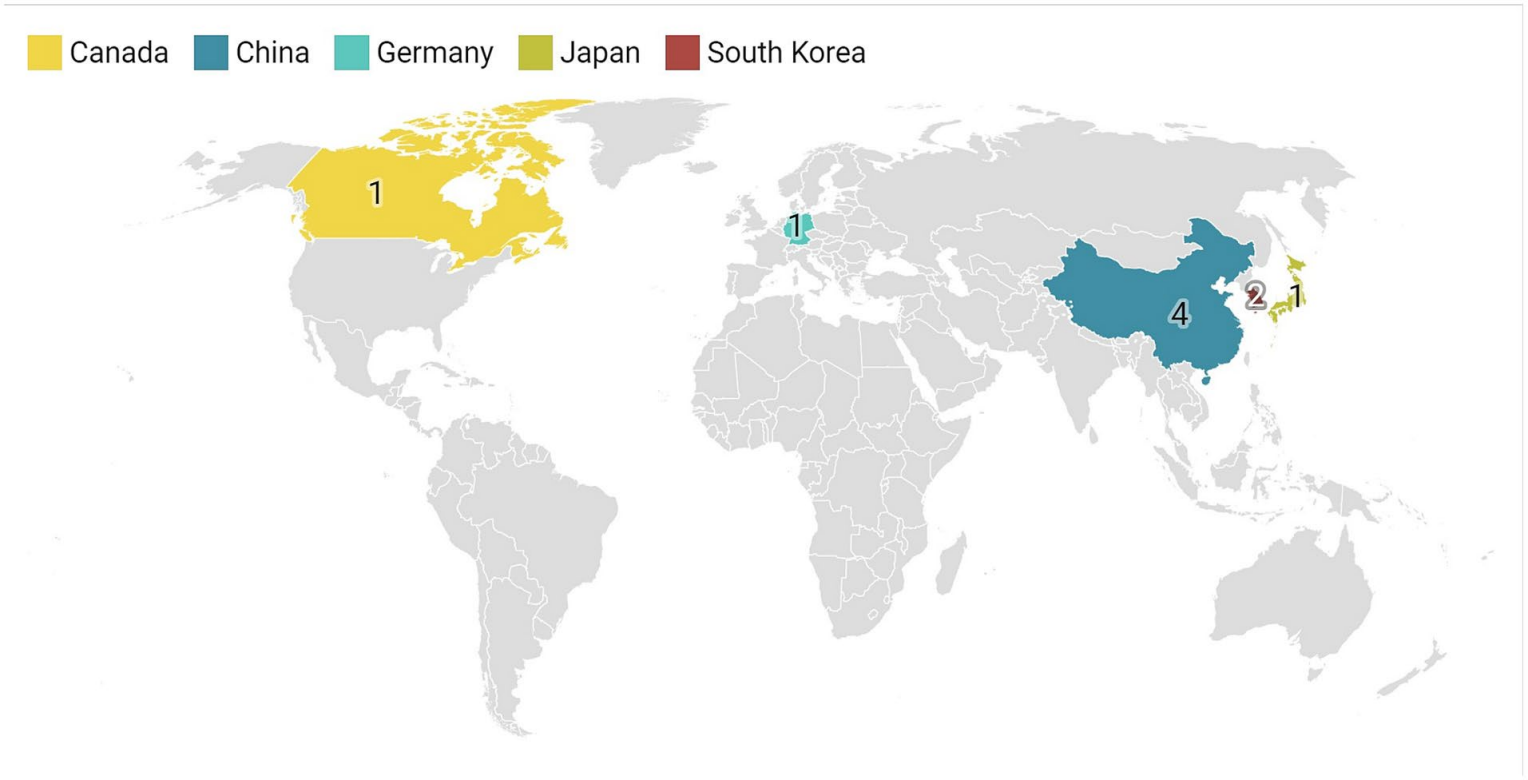
Shows the geographical distribution of the included studies.

This study analyzed studies conducted in Southeast Asian countries, with the majority from China (*n* = 4), South Korea (*n* = 2), and Japan (*n* = 1). Only one article each was from Germany and Canada (Figure 2). The findings of this study emphasize the importance of monitoring the occurrence of iron accumulation in the basal ganglia as a biomarker as well as the potential brain effects caused by the deposition of substances such as iron.

### 3.2 Meta-analysis and quality assessment results

**Figure 3:**
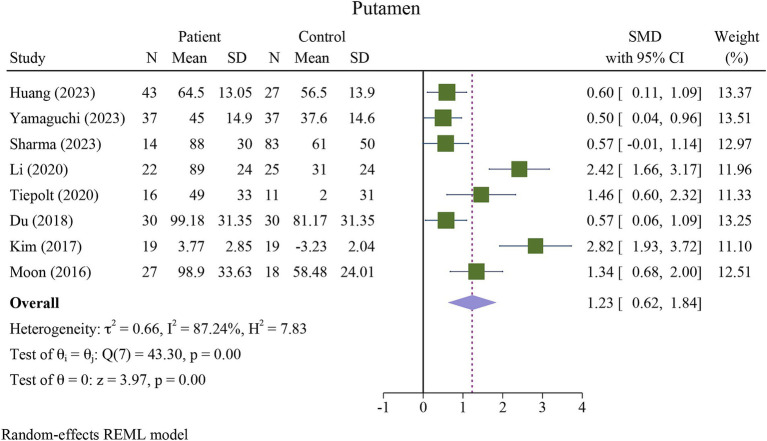
Meta-analysis of QSM values in the putamen (τ^2^: variance between studies, H index: the ratio of variance between studies to variance within studies, a measure of heterogeneity, Q index = Cochrane Q test, which is a heterogeneity statistical test; I^2^: a measure of the percentage of total variation across studies that is due to heterogeneity rather than chance. The test of θi = θ is a test for the homogeneity of effect sizes across different studies (θi and θj represent the effect sizes in two different studies). The test of θ = 0 was a test for the overall effect across all studies. Where θ represents the overall effect size).

**Figure 4:**
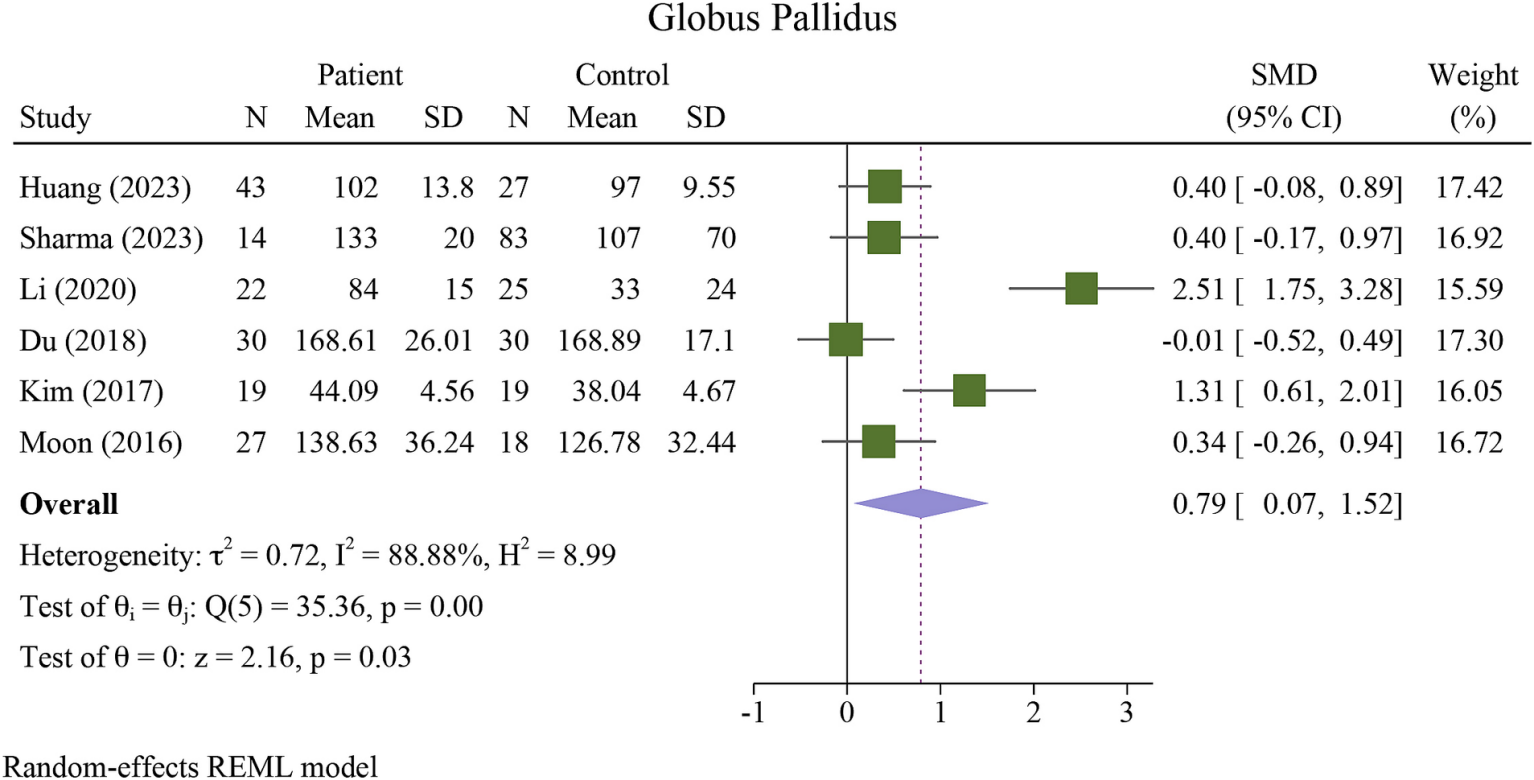
Meta-analysis of QSM values in the globus pallidus (τ^2^: variance between studies, H index: the ratio of variance between studies to variance within studies, a measure of heterogeneity, Q index = Cochrane Q test, which is a heterogeneity statistical test; I^2^: a measure of the percentage of total variation across studies that is due to heterogeneity rather than chance. The test of θi = θ is a test for the homogeneity of effect sizes across different studies (θi and θj represent the effect sizes in two different studies). The test of θ = 0 was a test for the overall effect across all studies. Where θ represents the overall effect size).

**Figure 5:**
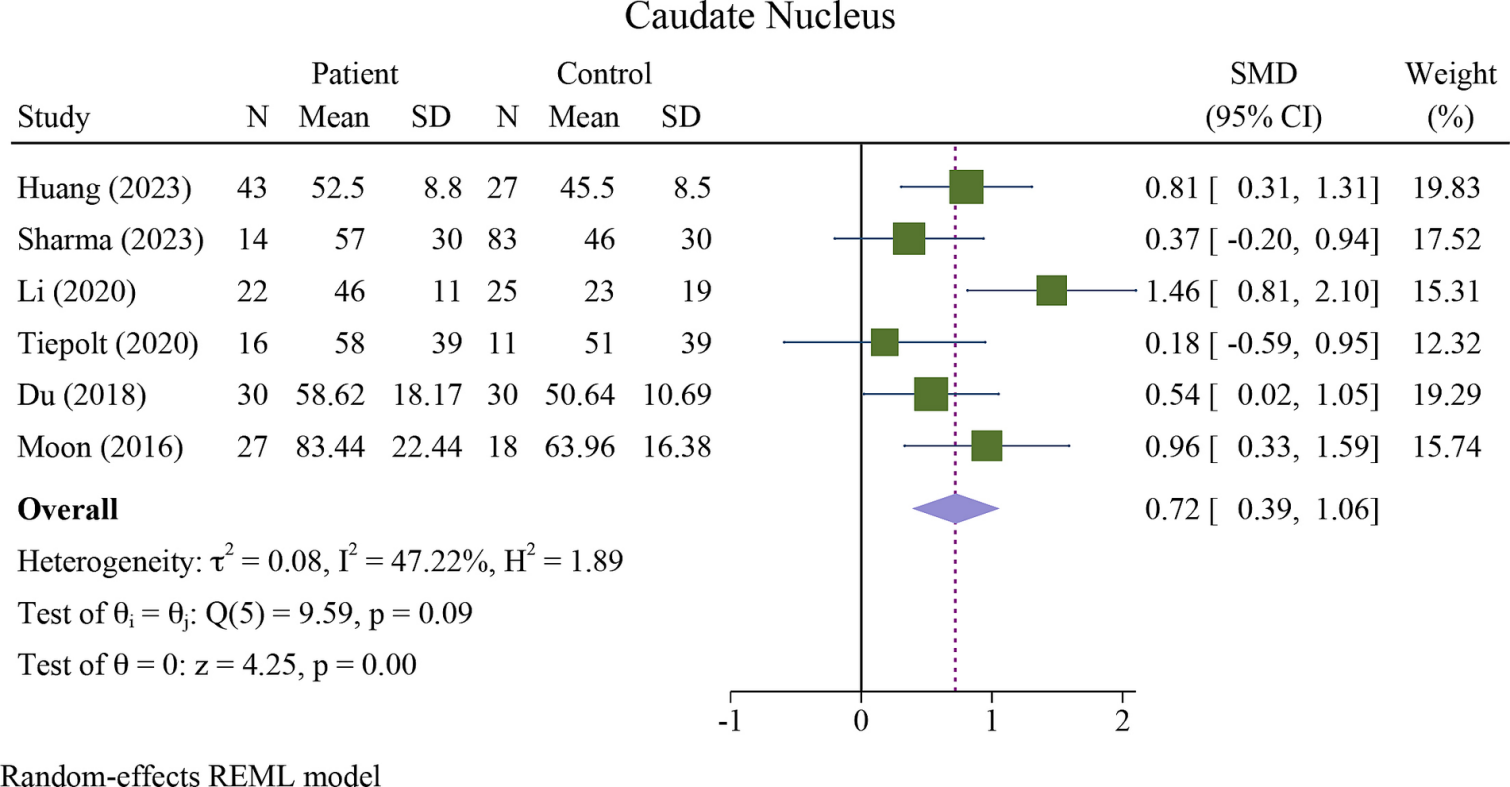
Meta-analysis of QSM values in the caudate nucleus (τ^2^: variance between studies, H index: the ratio of variance between studies to variance within studies, a measure of heterogeneity, Q index = Cochrane Q test, which is a heterogeneity statistical test; I^2^: a measure of the percentage of total variation across studies that is due to heterogeneity rather than chance. The test of θi = θ is a test for the homogeneity of effect sizes across different studies (θi and θj represent the effect sizes in two different studies). The test of θ = 0 was a test for the overall effect across all studies. Where θ represents the overall effect size).

**Figure 6:**
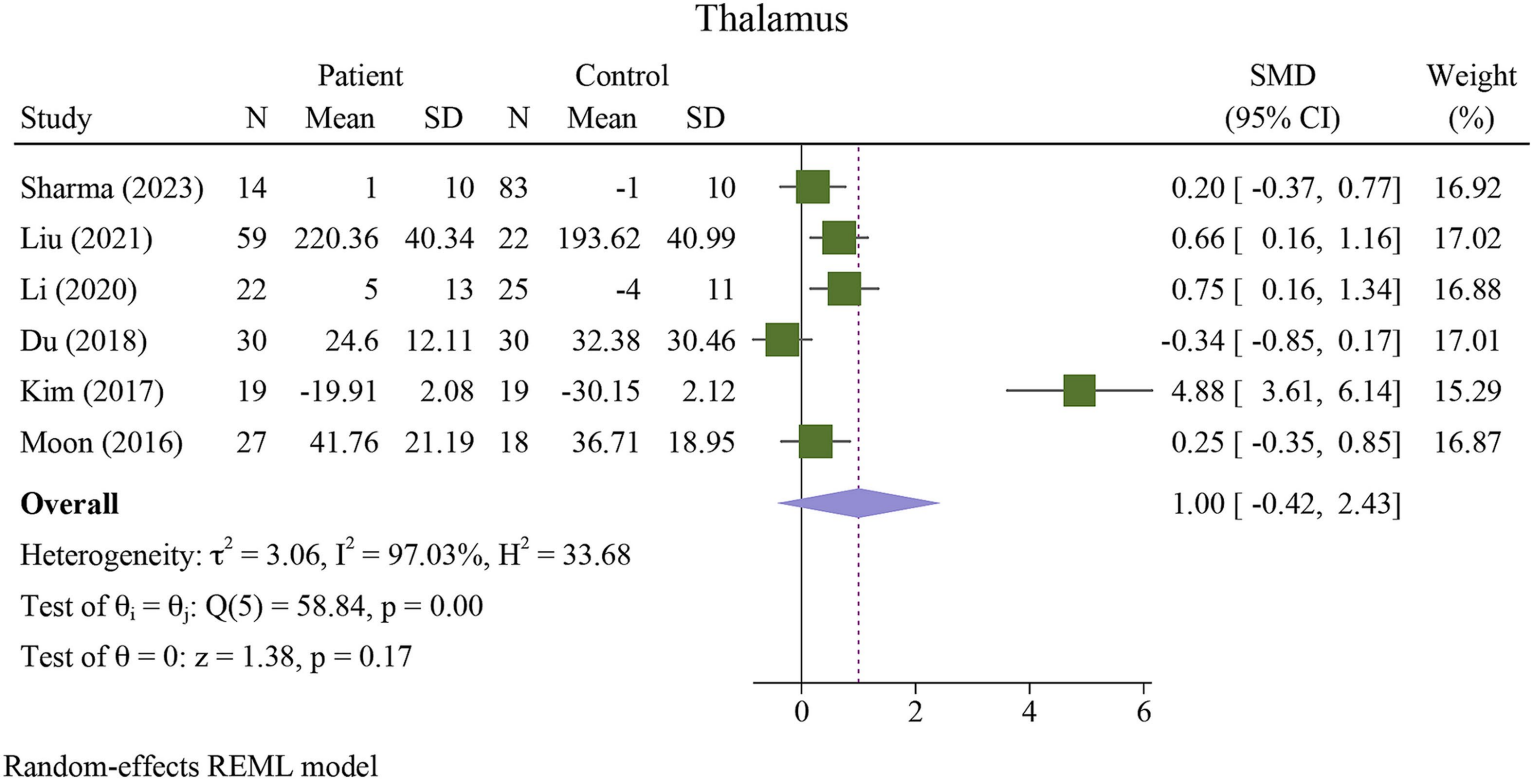
Meta-analysis of QSM values in the thalamus (τ^2^: variance between studies, H index: the ratio of variance between studies to variance within studies, a measure of heterogeneity, Q index = Cochrane Q test, which is a heterogeneity statistical test; I^2^: a measure of the percentage of total variation across studies that is due to heterogeneity rather than chance. The test of θi = θ is a test for the homogeneity of effect sizes across different studies (θi and θj represent the effect sizes in two different studies). The test of θ = 0 was a test for the overall effect across all studies. Where θ represents the overall effect size).

The meta-analysis showed a significant increase in iron deposition in the basal ganglia regions, such as the PUT, GP, and CN, as measured by QSM, when compared with HCs (Table 2). Specifically, the pooled SMD indicated a highly significant increase in iron in the PUT (SMD = 1.23, 95% CI = 0.62 to 1.84, *p* = 0.00, I^2^ = 87.24%, k = 8, *n* = 208) (Figure 3), nearly high increases in the GP (SMD = 0.79, 95% CI = 0.07 to 1.52, *p* = 0.03, I^2^ = 88.88%, k = 6, *n* = 155) (Figure 4), and moderate increases in the CN (SMD = 0.72, 95% CI = 0.39 to 1.06, *p* = 0.00, I^2^ = 47.22%, k = 6, *n* = 152) (Figure 5). However, there was no significant difference in the increase in iron deposition in the thalamus (SMD = 1.00, 95% CI = −0.42, 2.43, *p* = 0.17, I^2^ = 97.03%, k = 6, *n* = 171) between patients and controls (Figure 6).

**Table 2:** Meta-analysis results of QSM values in the basal ganglia nuclei of AD patients compared to healthy controls.

Brain region	SMD (95% CI)	*p*-value^a^	I^2^ (%)^b^/P_hetrogenity_	k^c^
Putamen	1.23 (0.62, 1.84)	0.00	87.24/0.00	8
Globus Pallidus	0.79 (0.07, 1.52)	0.03	88.88/0.00	6
Caudate Nucleus	0.72 (0.39, 1.06)	0.00	47.22/0.09	6
Thalamus	1.00 (−0.42, 2.43)	0.17	97.03/0.00	6

AD, Alzheimer’s disease; CI, confidence interval; QSM, quantitative susceptibility mapping; SMD, standardized mean difference.

a *p*-value < 0.05.

b A statistical measure of study heterogeneity.

c Number of studies.

There was severe heterogeneity between the included studies of PUT, GP, and thalamus, and moderate heterogeneity in the included studies of CN. Table 3 presents the details of the subgroup analysis carried out to further investigate heterogeneity (Supplementary Figures S1–S14). To explore potential sources of heterogeneity, we performed sensitivity and subgroup analyses. Our subgroup analysis indicated that age was a major source of heterogeneity in all studies on DGM nuclei (Table 3). Other factors, such as geographic distribution, ROB assessment, and sex, can contribute to heterogeneity, although to a lesser extent. Sensitivity analysis showed that none of the studies had a significant impact on the overall findings (Supplementary Figure S15).

**Table 3:** Subgroup analysis results of QSM values based on age and gender differences.

Brain region	Subgroup	SMD (95%CI)	*p*-value^a^	I^2^ (%)^b^/P_hetrogenity_	k^c^
Putamen	**Age**				
	≤ 69	0.68 (0.39, 0.96)	0.06	0.00/0.31	4
	> 69	1.73 (0.69, 2.77)		89.69/0.00	4
	**Sex (male%)**				
	≤ 30%	1.51 (0.61, 2.40)	0.41	84.70/0.00	4
	>30%	0.98 (0.11, 1.85)		89.66/0.00	4
	**Geographic Distribution**				
	Asia	1.33 (0.54, 2.12)	0.53	90.56/0.00	6
	Non Asia	0.95 (0.08, 1.82)		65.19/0.09	2
	**Risk of bias (ROB)**				
	High ROB	1.01 (0.15, 1.86)	0.46	88.91/0.00	4
	Low ROB	1.48 (0.55, 2.42)		86.35/0.00	4
Globus pallidus	**Age**				
	≤ 69	0.26 (−0.04, 0.56)	0.09	0.00/0.43	3
	> 69	1.37 (0.14, 2.60)		89.62//0.00	3
	**Sex (male%)**				
	≤ 30%	0.65 (0.08, 1.22)	0.72	64.21/0.07	3
	>30%	0.94 (−0.57, 2.46)		94.70/0.00	3
	**Risk of bias (ROB)**				
	High ROB	0.80 (−0.29, 1.88)	0.99	93.17//0.00	3
	Low ROB	0.81 (−0.14, 1.76)		76.44/0.04	3
Caudate nucleus	**Age**				
	≤ 69	0.53 (0.25, 0.81)	0.02	0.00/0.52	4
	> 69	1.20 (0.72, 1.69)		14.32/0.28	2
	**Sex (male%)**				
	≤ 30%	0.73 (0.38, 1.07)	0.91	0.00/0.28	3
	>30%	0.77 (0.13, 1.41)		73.24//0.03	3
	**Geographic distribution**				
	Asia	0.90 (0.54, 1.26)	0.04	38.61/0.18	4
	Non Asia	0.30 (−0.16, 0.76)		0.00/0.70	2
	**Risk of bias (ROB)**				
	High ROB	0.77 (0.34, 1.20)	0.71	58.94/0.07	3
	Low ROB	0.60 (−0.16, 1.37)		57.99//0.12	3
Thalamus	**Age**				
	≤ 69	−0.08 (−0.61, 0.44)	0.12	47.27/0.17	2
	> 69	1.57 (−0.48, 3.62)		97.46//0.00	4
	**Sex (male%)**				
	≤ 30%	2.53 (−2.01, 7.06)	0.34	97.61/0.00	2
	>30%	0.31 (−0.18, 0.81)		70.26//0.02	4
	**Risk of bias (ROB)**				
	High ROB	0.19 (−0.43, 0.81)	0.26	73.03/0.02	2
	Low ROB	1.88 (−0.97, 4.72)		98.00/0.00	4

AD, Alzheimer’s disease; CI, confidence interval; QSM, quantitative susceptibility mapping; SMD, standardized mean difference.

a Test of group differences, *p*-value < 0.05.

b A statistical measure of study heterogeneity.

c Number of studies.

Subgroup analysis based on age revealed that the difference in QSM values between patients with AD and HCs was more pronounced in the PUT and CN in the older age group (>69 years) (Supplementary Figures S1, S8). In contrast, there was no difference between the age subgroups of the GP and the thalamus (Supplementary Figures S5, S12).

Subgroup analysis showed that the increase in QSM values in the putamen was more pronounced in studies with a lower percentage of male participants (≤ 30%); however, in the CN group, it was more pronounced in studies with a higher percentage of male participants (> 30%) (Supplementary Figures S2, S9). Subgroup analysis based on sex also showed no significant differences in the QSM values between male and female patients with AD in the GP and thalamus (Supplementary Figures S6, S13).

Geographic distribution subgroup analysis revealed that the difference in QSM values between patients with AD and HCs was more pronounced in studies conducted in Asia than in non-Asian regions in the PUT (Supplementary Figure S3).

ROB assessment subgroup analysis revealed considerable differences in QSM values between studies with high ROB (SMD = 1.01, 95% CI = 0.15 to 1.86) and low ROB (SMD = 1.45, 95% CI = 0.55 to 2.42) for PUT (Supplementary Figure S4).

### 3.3 Publication bias analysis

**Figure 7:**
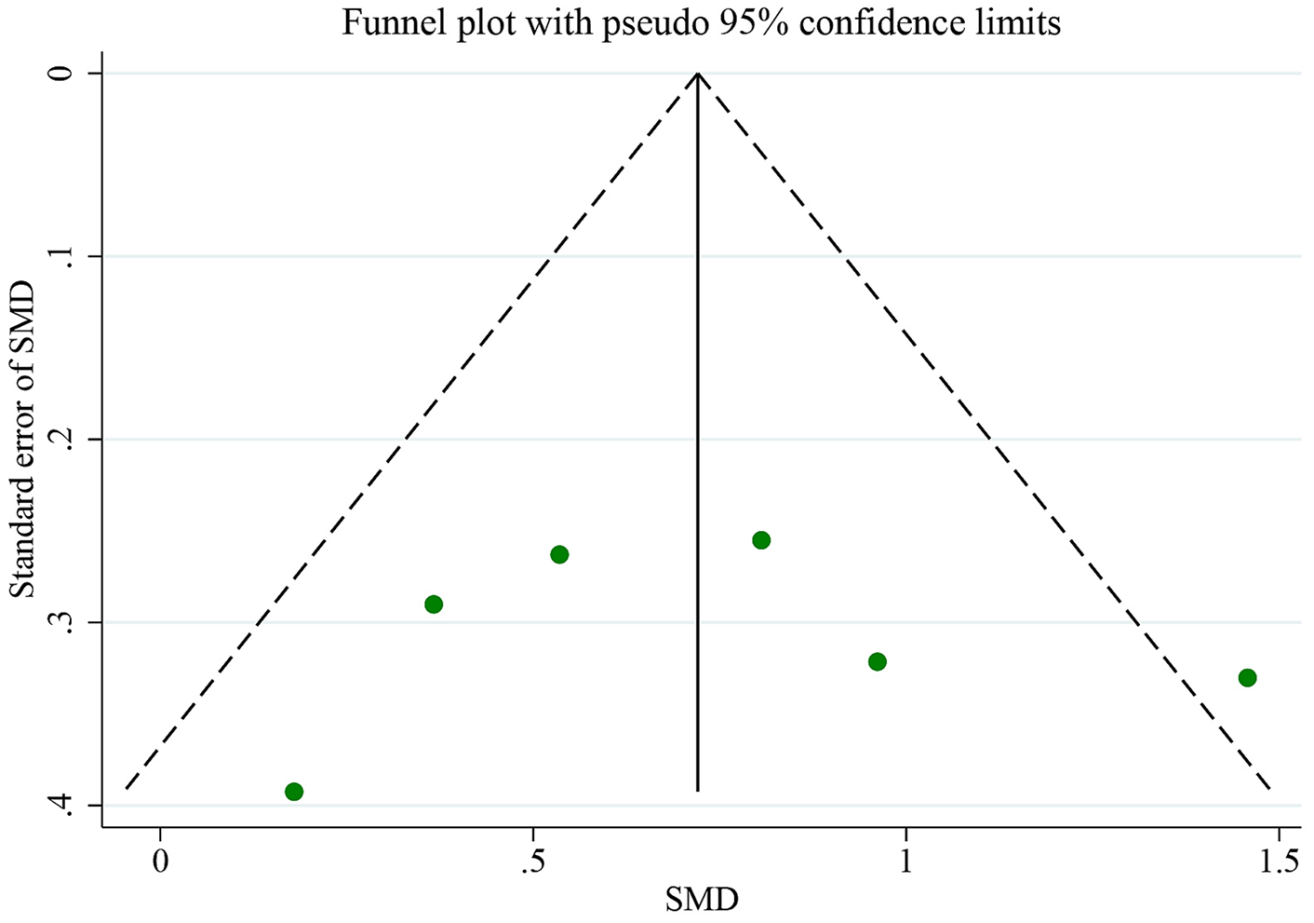
The assessments for publication bias include funnel plots.

The QSM technique and comorbidity studies were used to examine the iron levels in the PUT. Figure 7 displays a funnel plot, which indicates no publication bias, a finding that was confirmed by Egger’s test (*p* = 0.632). To evaluate publication bias, the authors employed Egger’s test, with *p* < 0.05 indicating significant publication bias. The authors conducted a linear regression analysis to analyze publication bias, which involved intercept and slope parameters. The formula used to calculate this was yi = a + βxi + ϵi (Ávila-Villanueva et al., 2022) i = 1… r (r = the number of studies), where yi represents the standardized estimate, xi signifies the precision of studies, and ϵi denotes the error term.

## 4 Discussion

The study demonstrated a significant pathological increase in iron deposition in the basal ganglia, specifically in the PUT, GP, and CN of patients with AD compared to HCs. These results are consistent with the growing body of literature suggesting that QSM is a key imaging biomarker for detecting iron accumulation in the brain in the pathogenesis of neurodegenerative diseases, such as AD (van Bergen et al., 2016; Young et al., 2020; Ravanfar et al., 2021; Uchida et al., 2022; Cogswell and Fan, 2023; Ghaderi et al., 2023).

Neurodegenerative diseases such as AD and Parkinson’s disease (PD) are primarily associated with aging. AD affects one in ten individuals aged 65 years or older, and its prevalence increases with age (Hou et al., 2019). Our study showed that the PUT appears to be the most potentially susceptible region, showing distinct iron increases detectable with QSM, which is consistent with a recent study on the deep gray matter nuclei of a healthy aging population, where they are most prominent in the putamen (Madden and Merenstein, 2023). The substantial increase in the QSM values in the putamen supports the hypothesis that this region is particularly vulnerable to iron dysregulation in AD. The putamen is involved in both motor and cognitive functions, both of which are impaired in AD. The role of iron in facilitating oxidative stress, inflammation, and aggregation of amyloid-beta and tau proteins could explain the association between increased iron levels in the putamen and the progression of AD (Ward et al., 2014; Galaris et al., 2019; Ndayisaba et al., 2019; Yan and Zhang, 2020). In contrast, it is worth noting that the thalamus did not show a significant difference in QSM values between AD patients and controls, which may indicate regional specificity in the brain’s iron distribution related to AD (Moon et al., 2016; Du et al., 2018; You et al., 2021; Sharma et al., 2023).

As a whole, the specific deposition of iron may be related to the susceptibility of different brain regions to oxidative stress and the progression of neurodegenerative diseases (Schipper, 2012; Ward et al., 2014; Bulk et al., 2018; Guan et al., 2022). Further research involving larger studies is necessary to elucidate the role of iron deposition characteristics of DGM nuclei in AD.

Heterogeneity is a limitation that must be considered when interpreting these results. The high heterogeneity among the studies suggests variability in factors such as age, sex, geographic distribution, and ROB assessment. The included studies on DGM nuclei have shown that age is a major source of heterogeneity. In addition to age, other factors, such as geographic distribution, ROB assessment, and sex can also contribute to heterogeneity, although their impact is relatively low.

It is important to note that the *p*-value can be significantly affected by the number of studies included in the subgroup meta-analysis. When there were five or fewer studies in a subgroup, the *p* value tended to be non-significant. To determine the difference between the effect sizes (ES) of the subgroups, we used a 50% overlapping CI. If the CI overlaps by more than 50% between subgroups, it indicates that the difference between the SMDs is considerable. Our study found no statistically significant differences in the QSM value of PUT between subgroups based on age, sex, geographic distribution, and ROB. Similarly, there were no significant differences in the QSM value of CN between the sex subgroups (*p* > 0.05). However, we observed a considerable difference in SMDs using a 50% overlap of CI.

Subgroup analyses revealed that older age (>69 years) in PUT and CN, as well as a lower percentage of male subjects (≤30%) in PUT, were associated with greater iron accumulation in AD patients than in HCs. However, a higher percentage of male subjects (>30%) in the CN group were more susceptible to iron deposition. This highlights the impact of demographic factors on the regional iron pathology in patients with AD (Tran et al., 2022). These results indicate that age and sex may play a role in the variability of iron deposition in specific basal ganglia nuclei (Ficiarà et al., 2022; Li et al., 2023). This aligns with studies showing an age-related increase in brain iron levels (Bartzokis et al., 2011) and variability in the prevalence of iron overload in AD among sex-based features (Altmann et al., 2014).

The larger effect observed in the QSM value of PUT in Asian populations could point toward genetic or environmental factors that modulate iron accumulation in AD. Our findings are consistent with a recent study that was conducted to investigate the diagnostic value of DGM magnetic susceptibility in AD in China (Huang et al., 2023). The study aimed to analyze differences in QSM values among 93 subjects and correlate the findings with neuropsychiatric scales. The results indicated that magnetic susceptibility values in the bilateral caudate nucleus and right putamen were significantly higher in AD patients and those with mild cognitive impairment than in healthy controls. The study also found that significant differences were present in more regions among Apolipoprotein E epsilon4 (APOE-ε4) non-carriers. These findings suggest that investigating the correlation between deep gray matter iron levels and AD could provide valuable insights into AD pathogenesis and facilitate early diagnosis in elderly Chinese. However, these results should be interpreted with caution because of potential confounding factors, and should be elucidated in future studies. Furthermore, the impact of study quality on the results was also evident, as studies with an ROB showed lower SMD than those with a low ROB.

The presence of excess iron in the basal ganglia of patients with AD aligns with previous studies that used both postmortem tissue analysis and *in vivo* MRI techniques (Langkammer et al., 2012; Acosta-Cabronero et al., 2013). There are several potential reasons for this accumulation. Iron is a metal that can participate in Fenton reactions, generating reactive oxygen species (ROS) and causing oxidative stress (Zhao, 2019). Consequently, increased iron levels in the basal ganglia may contribute to the observed neuronal damage and synaptic loss in AD (Peters et al., 2015; Belaidi and Bush, 2016). Additionally, iron plays a role in dopamine metabolism, a neurotransmitter that is downregulated in AD. Therefore, disruption of iron homeostasis could impact dopamine metabolism and contribute to the motor and cognitive symptoms associated with AD (Hare et al., 2013; Ferreira et al., 2019; Wojtunik-Kulesza et al., 2019). Finally, iron is necessary for the production of myelin, a lipid-rich substance that insulates neurons and aids in the transmission of electrical signals (Stadelmann et al., 2019). The decreased production of myelin in AD may be connected to the heightened iron deposition in the basal ganglia (Liu et al., 2018; Khattar et al., 2021; Tran et al., 2022).

While the mechanism linking iron accumulation to AD remains unclear, this meta-analysis provides strong evidence that excessive iron deposition occurs preferentially in the basal ganglia of brains with AD. Given that iron is implicated in oxidative stress and amyloidogenesis, elevated iron levels may contribute to neurodegeneration through multiple pathogenic pathways in AD. Prospective studies are needed to determine if basal ganglia QSM measures can serve as early biomarkers for disease progression or therapeutic monitoring in AD.

It is important to note that this meta-analysis has some limitations. The included studies only provide a cross-sectional comparison between AD patients and healthy controls, which means that we cannot determine whether elevated subcortical iron levels occur before or after the onset of AD. Without longitudinal data tracking changes in iron deposition over time, the causal link between excessive iron and the progression of AD cannot be established. To further validate QSM as a reliable biomarker, more high-quality research with standardized protocols is needed (QSM Consensus Organization Committee et al., 2024). Specifically, longitudinal cohort studies that use regular QSM in at-risk populations before disease onset could help establish causative links between excessive iron and AD progression. Furthermore, examining QSM changes before and after iron reduction therapies may clarify the potential of this biomarker for therapeutic monitoring. Finally, multicenter collaborations with harmonized QSM acquisition and analysis would greatly enhance sample sizes and generalizability. Overall, larger prospective studies tracking within-patient fluctuations in regional brain iron over time are needed to fully capture the evolving role of iron dyshomeostasis in AD and the promise of QSM as a marker of disease state.

## 5 Conclusion

This study provides evidence that QSM is a promising tool for quantifying iron in the basal ganglia as a potential biomarker of AD. The significant increase in iron levels in the PUT, GP, and CN of patients with AD suggests the involvement of iron dysregulation in AD pathophysiology. Age, sex, geographic distribution, and study quality were found to influence iron accumulation in specific basal ganglia nuclei. Further longitudinal studies with standardized methodologies are required to establish causal relationships and explore the utility of QSM in early diagnosis and monitoring of AD progression.

## Data availability statement

The original contributions presented in the study are included in the article/Supplementary material, further inquiries can be directed to the corresponding author.

## Author contributions

SG: Conceptualization, Data curation, Formal analysis, Investigation, Methodology, Project administration, Resources, Software, Supervision, Validation, Visualization, Writing – original draft, Writing – review & editing. SM: Conceptualization, Data curation, Formal analysis, Investigation, Methodology, Project administration, Resources, Software, Supervision, Validation, Visualization, Writing – original draft, Writing – review & editing. NN: Data curation, Investigation, Writing – original draft. SK: Data curation, Investigation, Writing – original draft. FS: Formal analysis, Software, Validation, Writing – original draft.
