# Supplementary material for: Iron quantification in basal ganglia: quantitative susceptibility mapping as a potential biomarker for Alzheimer’s disease – a systematic review and meta-analysis
Source: Front Neurosci. 2024 Feb 26;18:1338891. doi: 10.3389/fnins.2024.1338891 (PMC10925682; doi:10.3389/fnins.2024.1338891)
Supplement: Supplementary file 1 [file Table_2.DOCX]

| **Table S1.** The search strategies used for database searches | |
| --- | --- |
| **Database** | **N** |
| PubMed/Medline: ("Alzheimer's disease") AND ("quantitative susceptibility mapping" OR "QSM") AND ("basal ganglia" OR "Striatum" OR "caudate nucleus" OR "putamen" OR "globus pallidus" OR "substantia nigra pars reticulata" OR "subthalamic nucleus" OR "thalamus" OR "red nucleus" OR "substantia nigra pars compacta" OR "substantia nigra") | 27 |
| Scopus: TITLE-ABS-KEY ( "Alzheimer's disease" ) AND TITLE-ABS-KEY ( "quantitative susceptibility mapping" OR "QSM" ) AND TITLE-ABS-KEY ( "basal ganglia" OR "striatum" OR "caudate nucleus" OR "putamen" OR "globus pallidus" OR "subthalamic nucleus" OR "thalamus" OR "red nucleus" OR "substantia nigra" ) | 38 |
| Web of Science: TS=(("Alzheimer's disease") AND ("quantitative susceptibility mapping" OR "QSM") AND ("basal ganglia" OR "Striatum" OR "caudate nucleus" OR "putamen" OR "globus pallidus" OR "subthalamic nucleus" OR "thalamus" OR "red nucleus" OR "substantia nigra")) | 37 |
| Google Scholar: “"quantitative susceptibility mapping" "Alzheimer's disease" "basal ganglia" "striatum" "caudate nucleus" "putamen" "globus pallidus" "subthalamic nucleus" "thalamus" "Red nucleus" "substantia nigra" "basal ganglia" OR "Striatum" OR "caudate nucleus" OR "putamen" OR "globus pallidus" OR "subthalamic nucleus" OR "thalamus" OR "red nucleus" OR "Substantia nigra" "QSM"” | 35 |


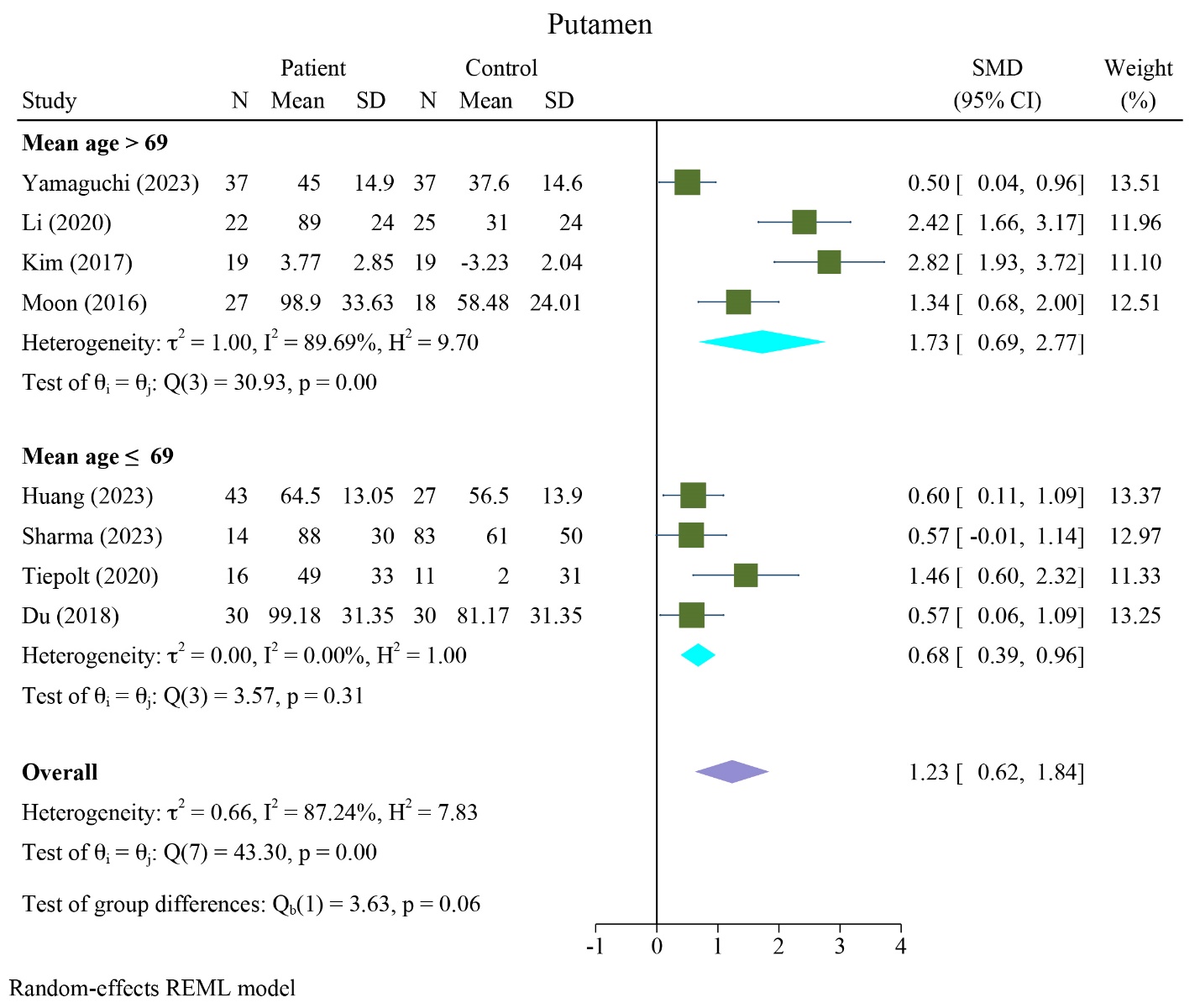


**FIGURE S1** Subgroup analysis results of QSM values based on age differences in the putamen


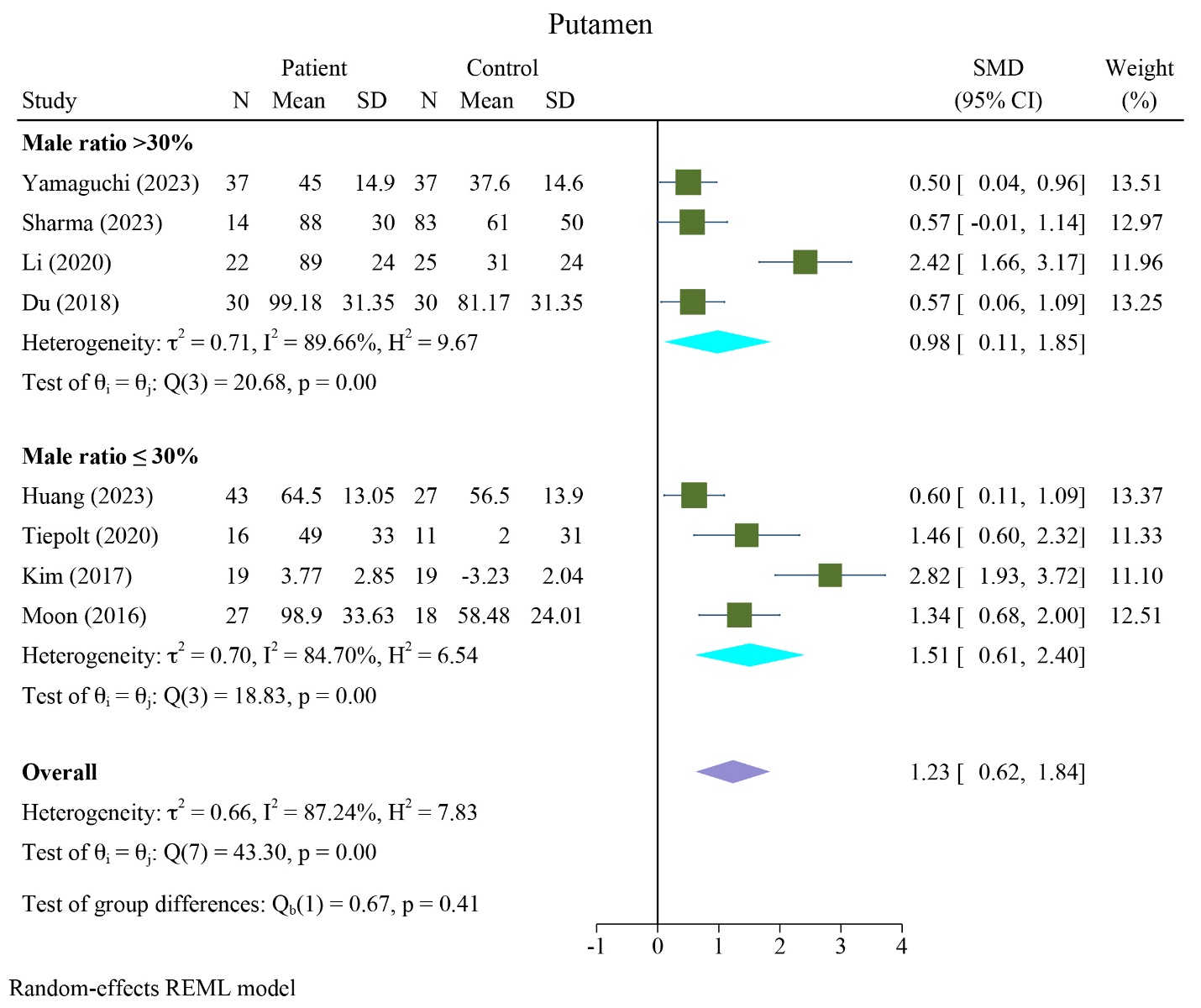


**FIGURE S2** Subgroup analysis results of QSM values based on sex differences in the putamen


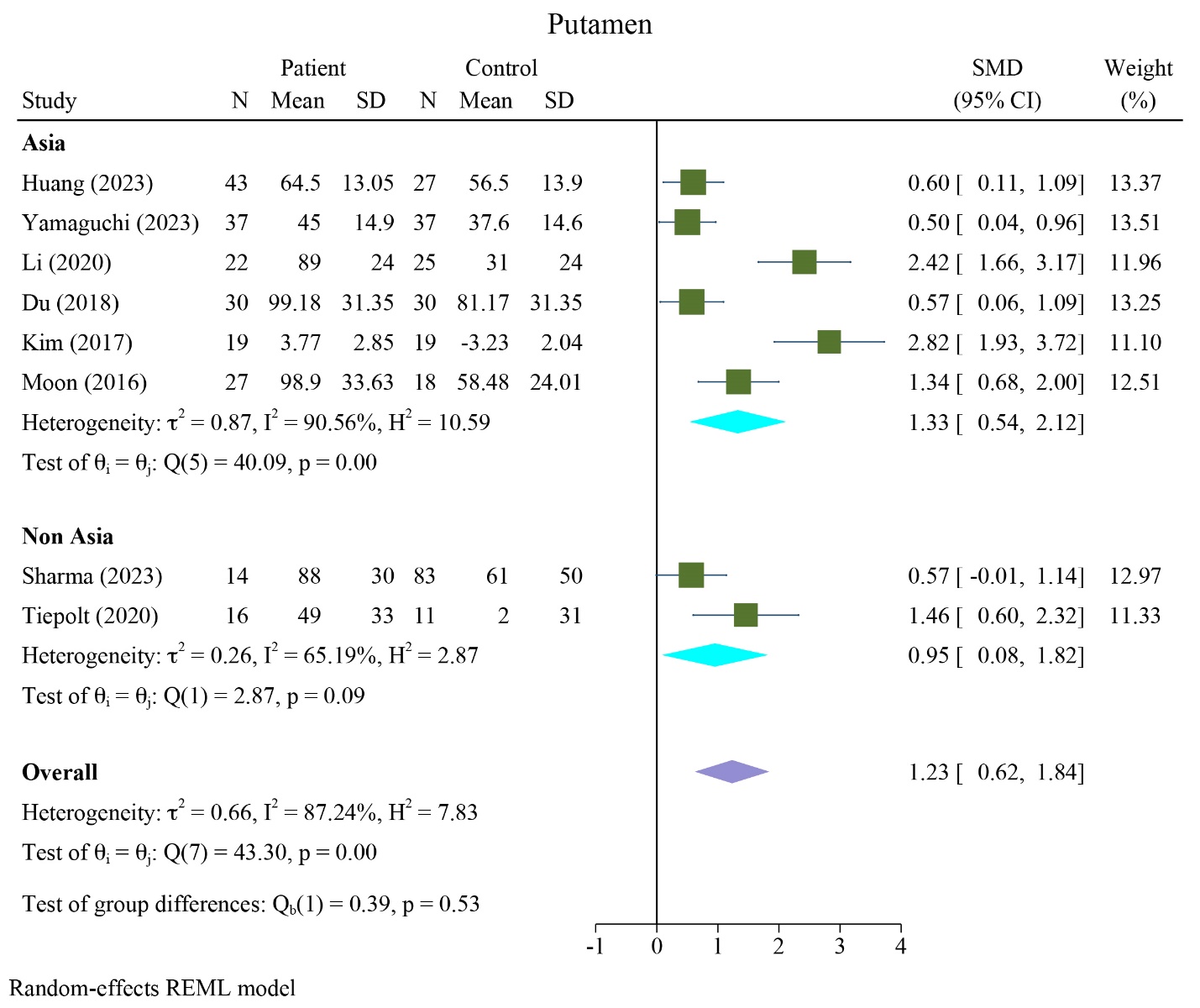


**FIGURE S3** Subgroup analysis results of QSM values based on region differences in the putamen


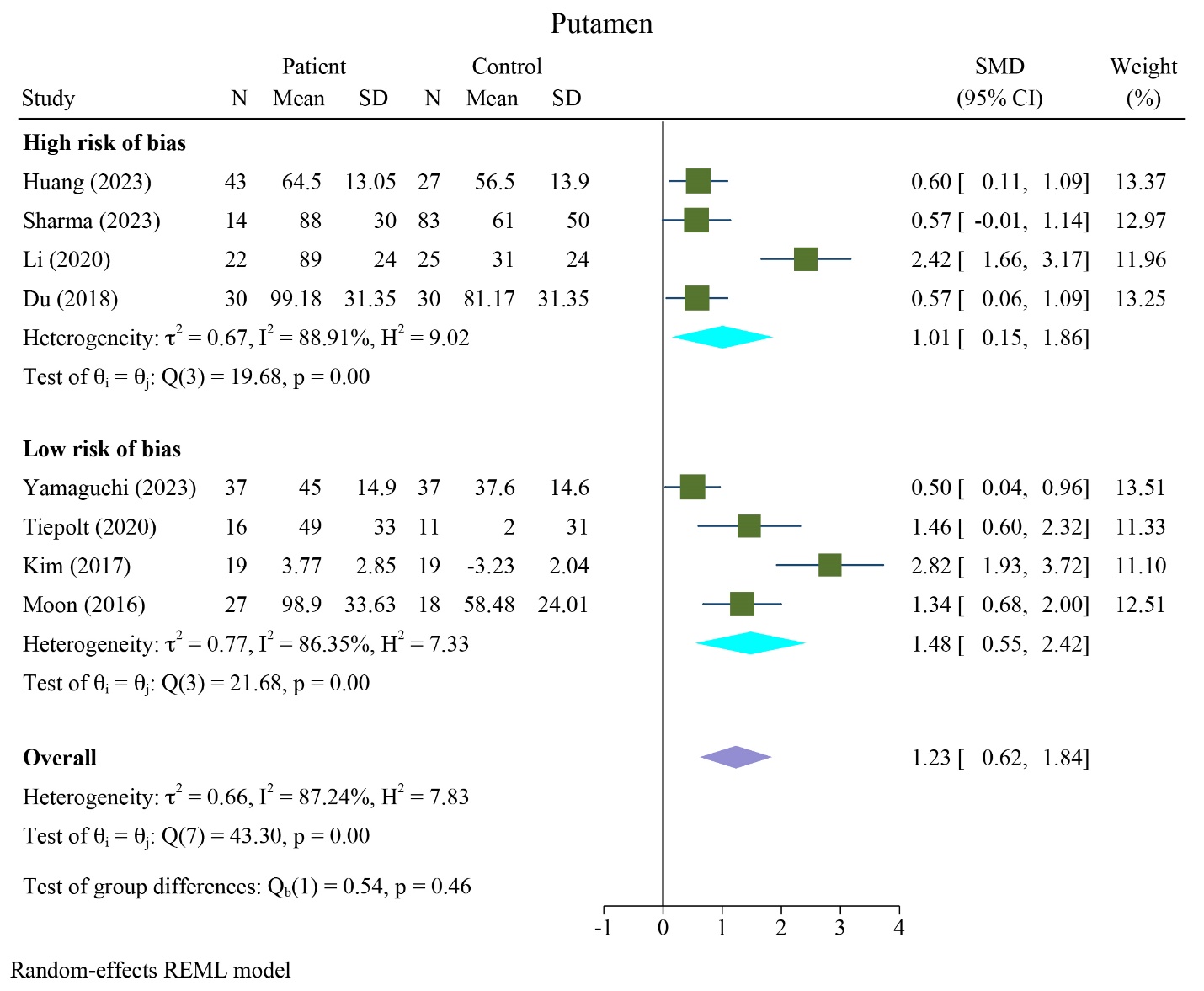


**FIGURE S4** Subgroup analysis results of QSM values based on ROB assessments in the putamen


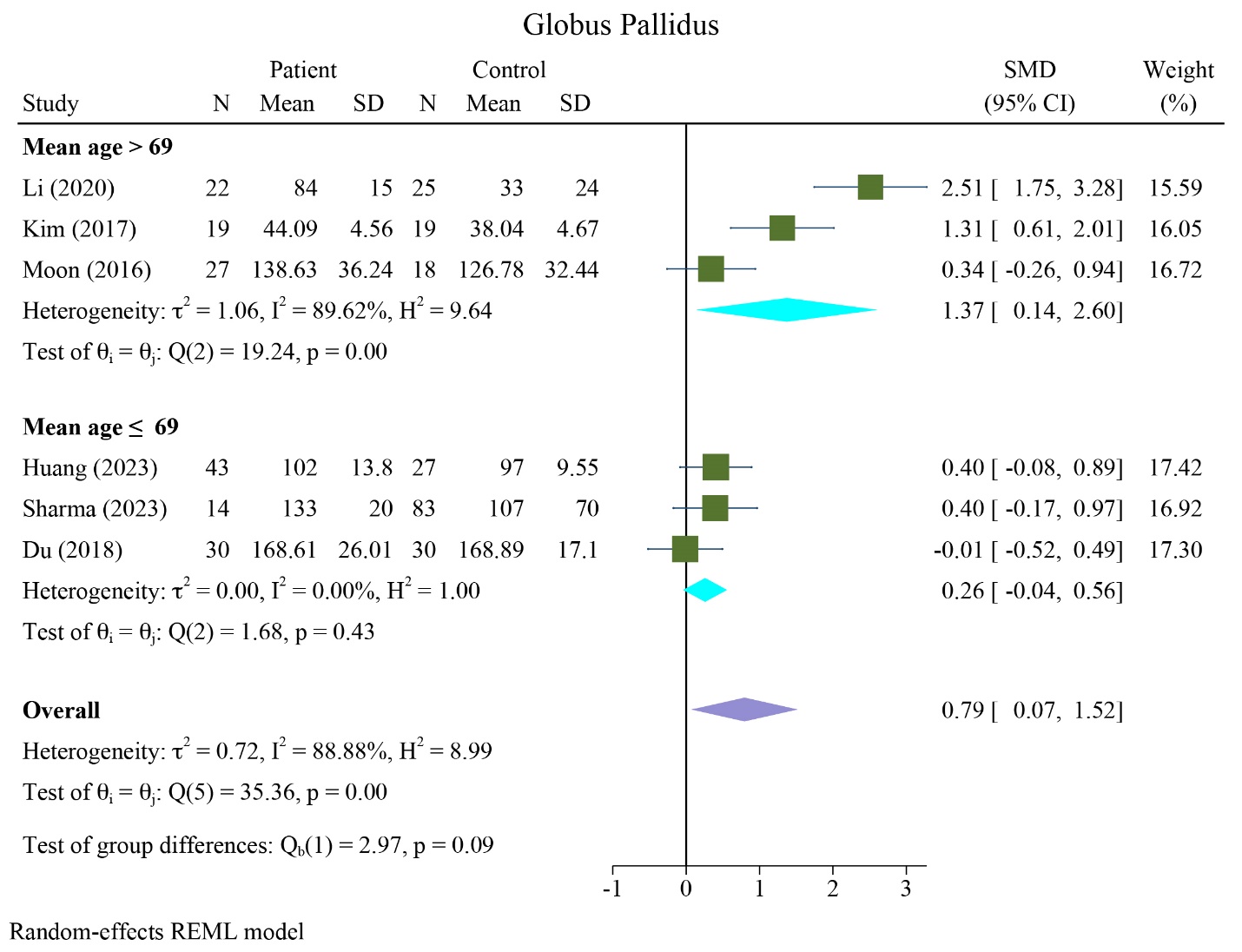


**FIGURE S5** Subgroup analysis results of QSM values based on age differences in the globus pallidus


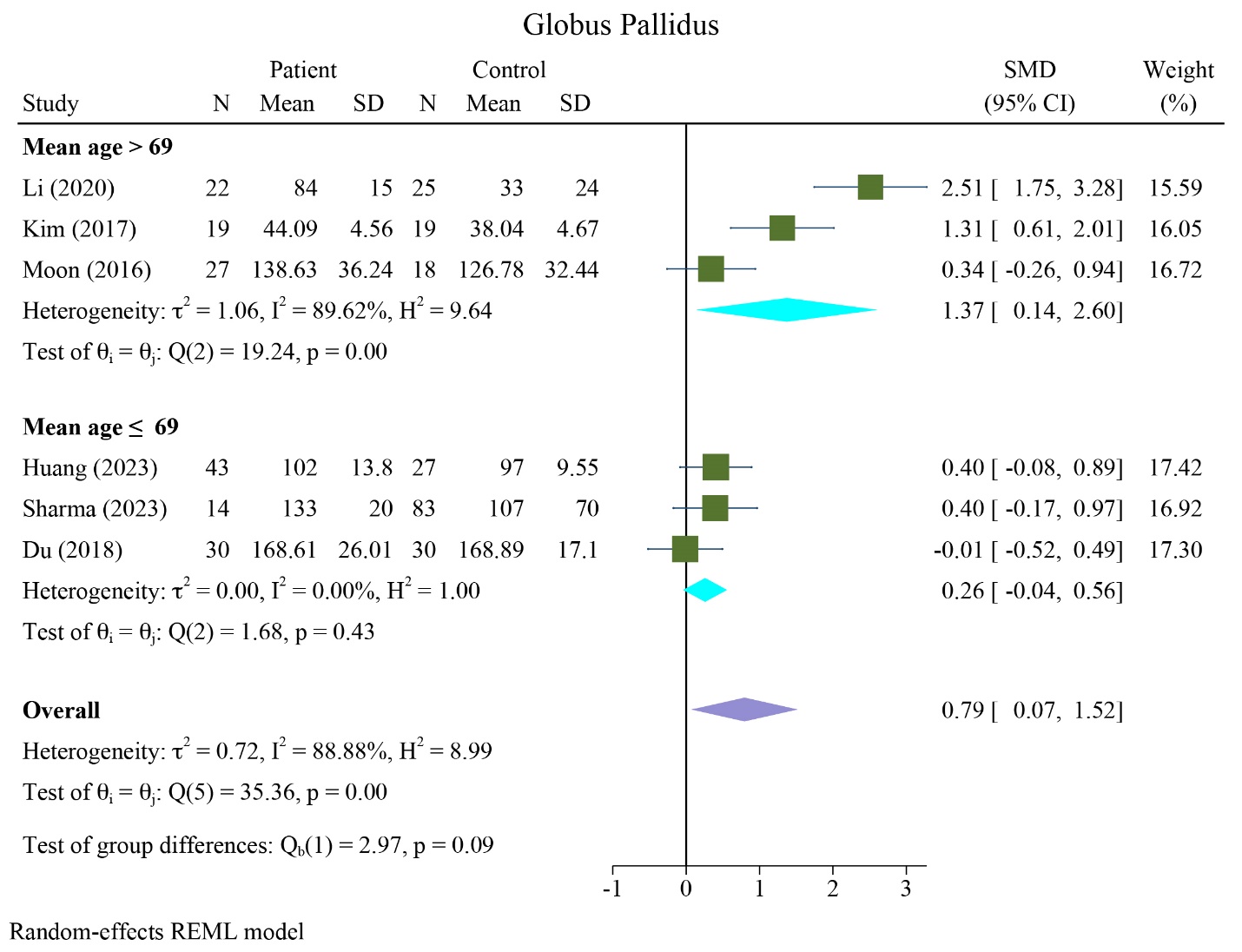


**FIGURE S6** Subgroup analysis results of QSM values based on sex differences in the globus pallidus


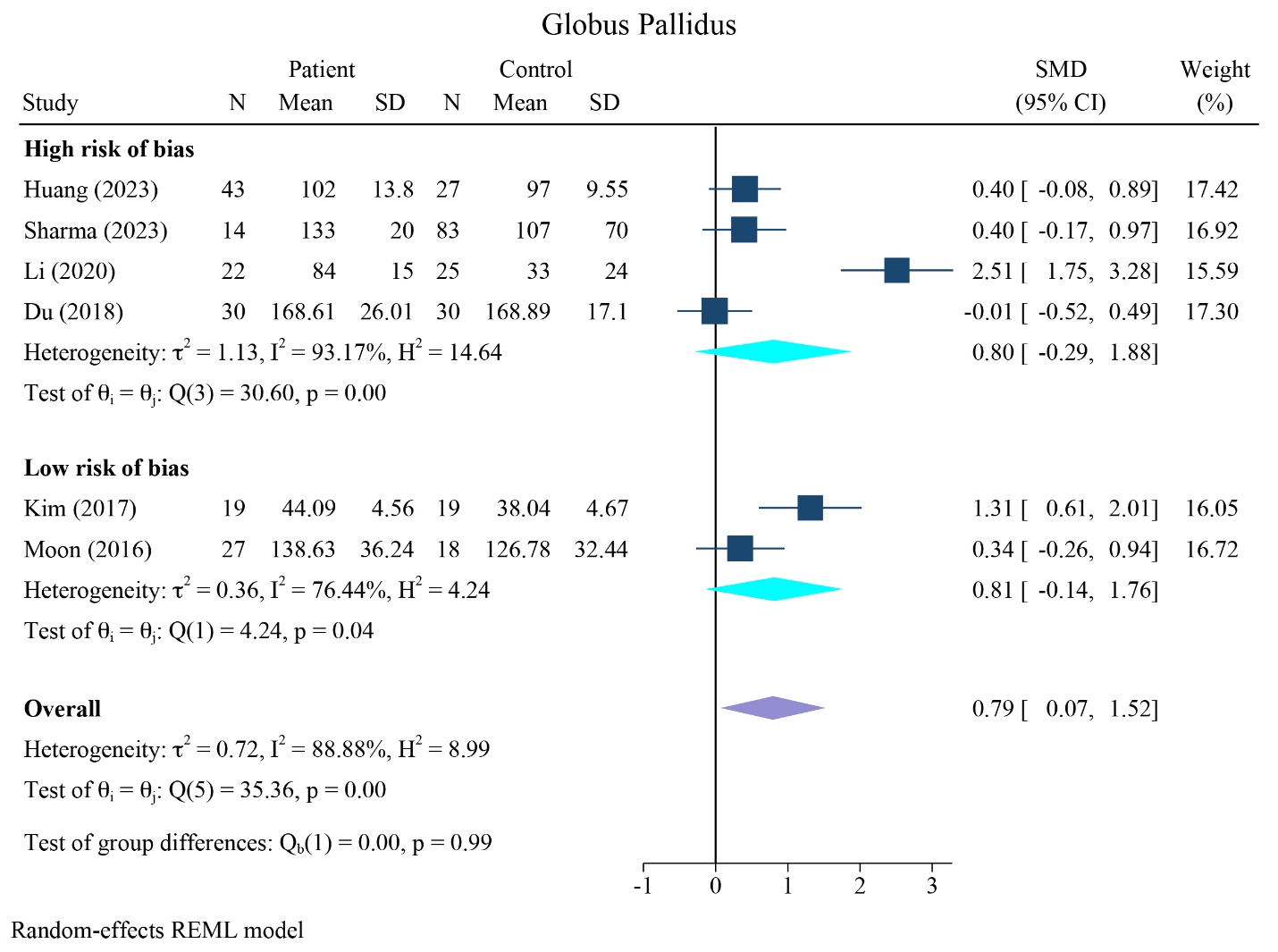


**FIGURE S7** Subgroup analysis results of QSM values based on ROB assessments in the putamen


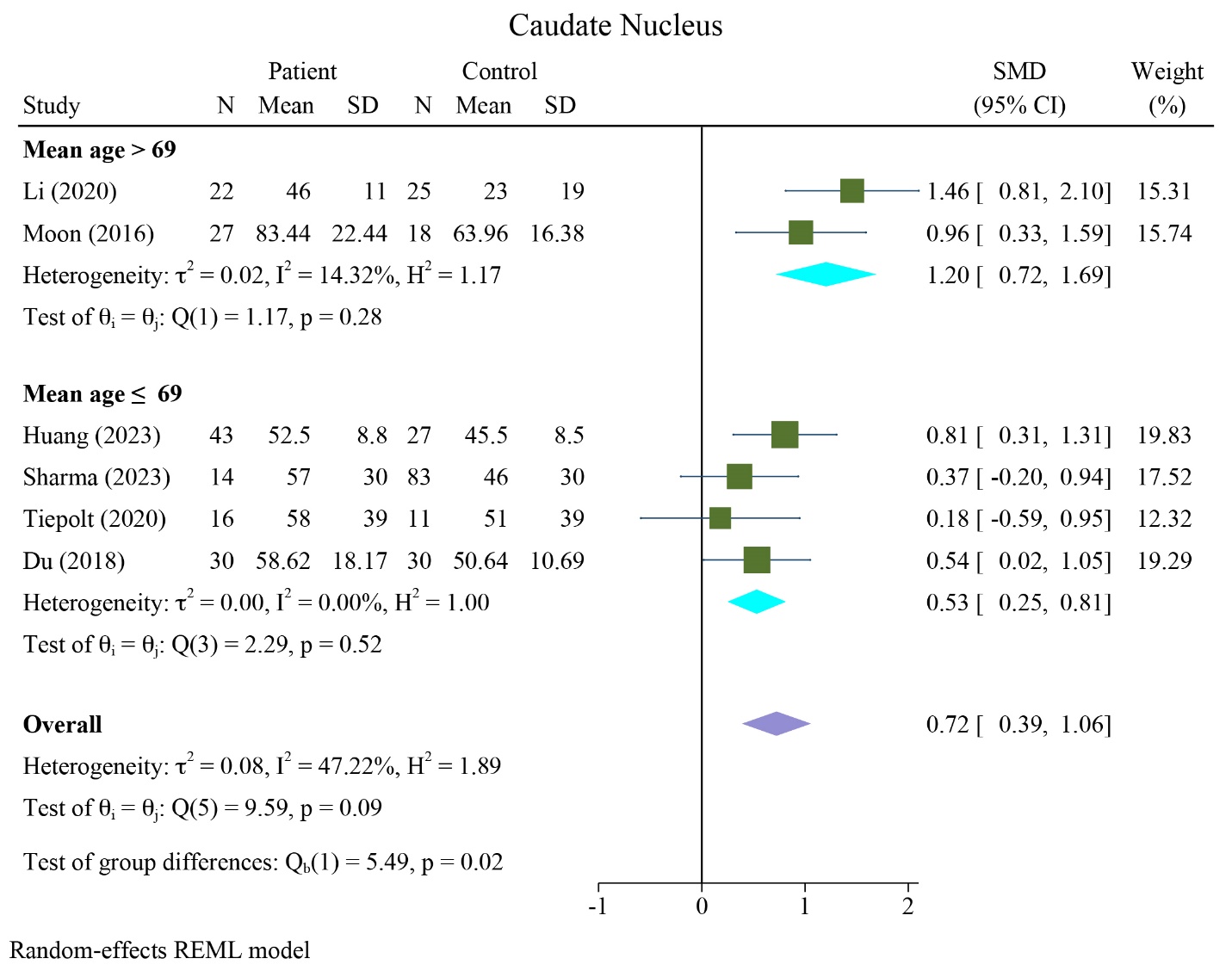


**FIGURE S8** Subgroup analysis results of QSM values based on age differences in the caudate nucleus


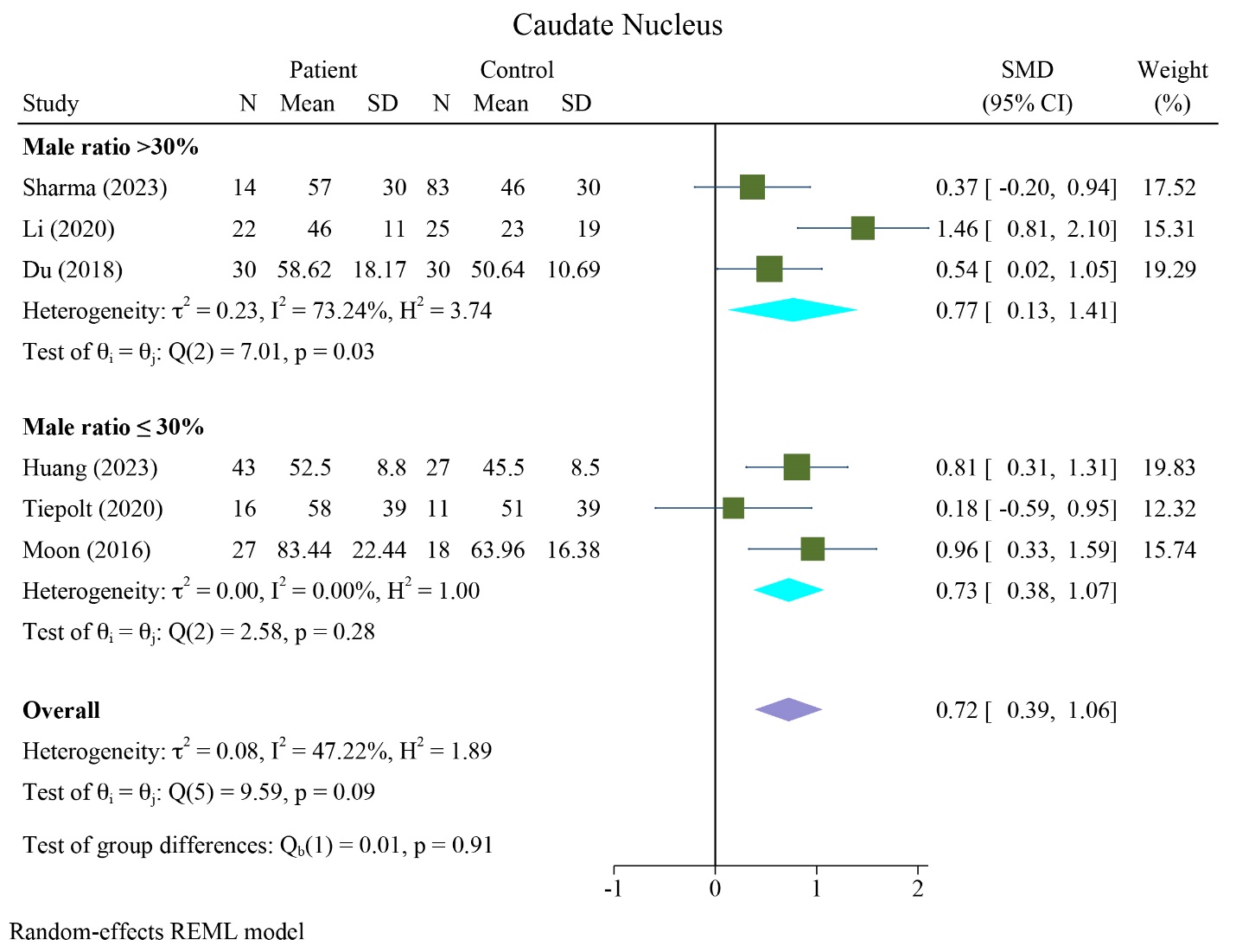


**FIGURE S9** Subgroup analysis results of QSM values based on sex differences in the caudate nucleus


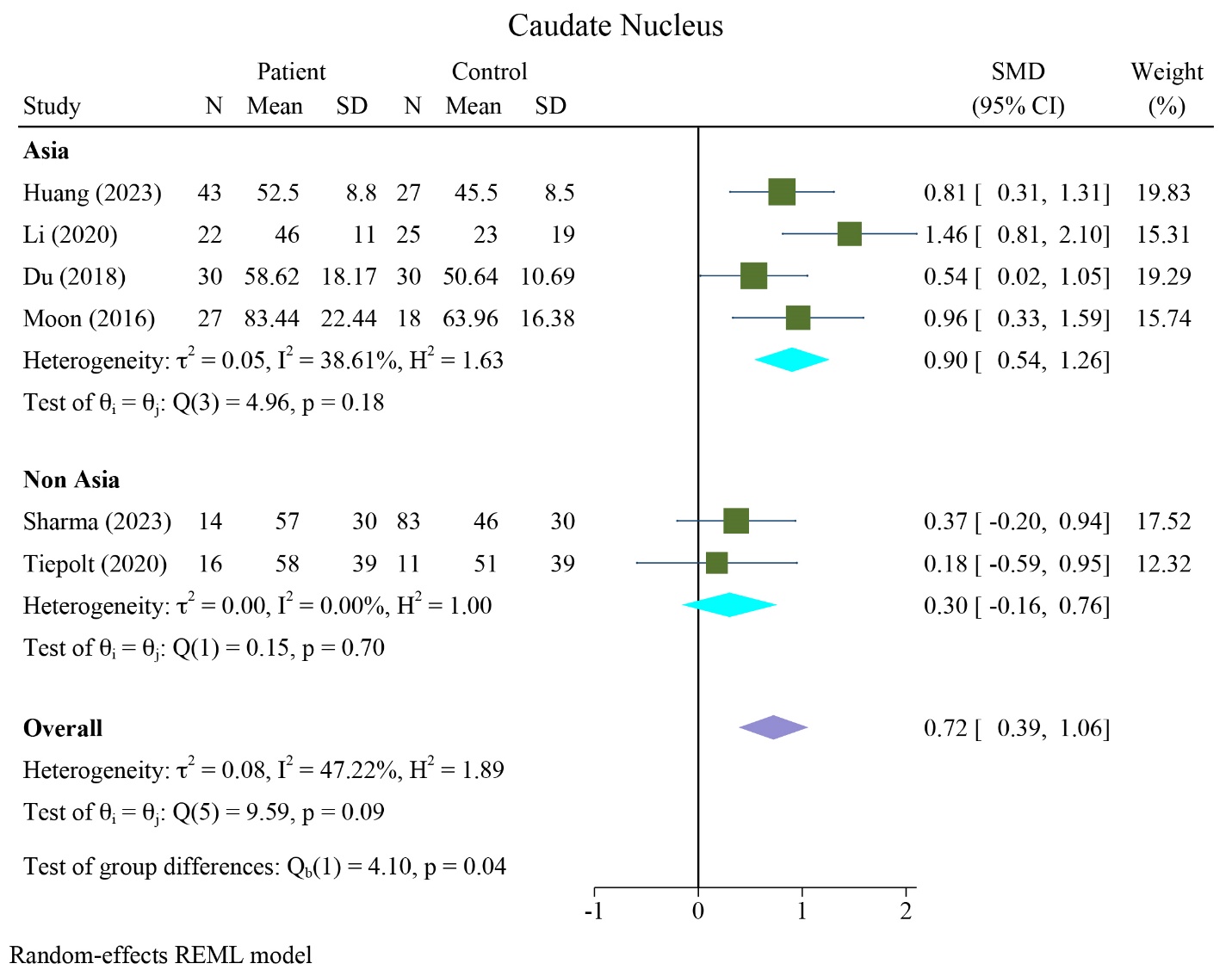


**FIGURE S10** Subgroup analysis results of QSM values based on region differences in the caudate nucleus


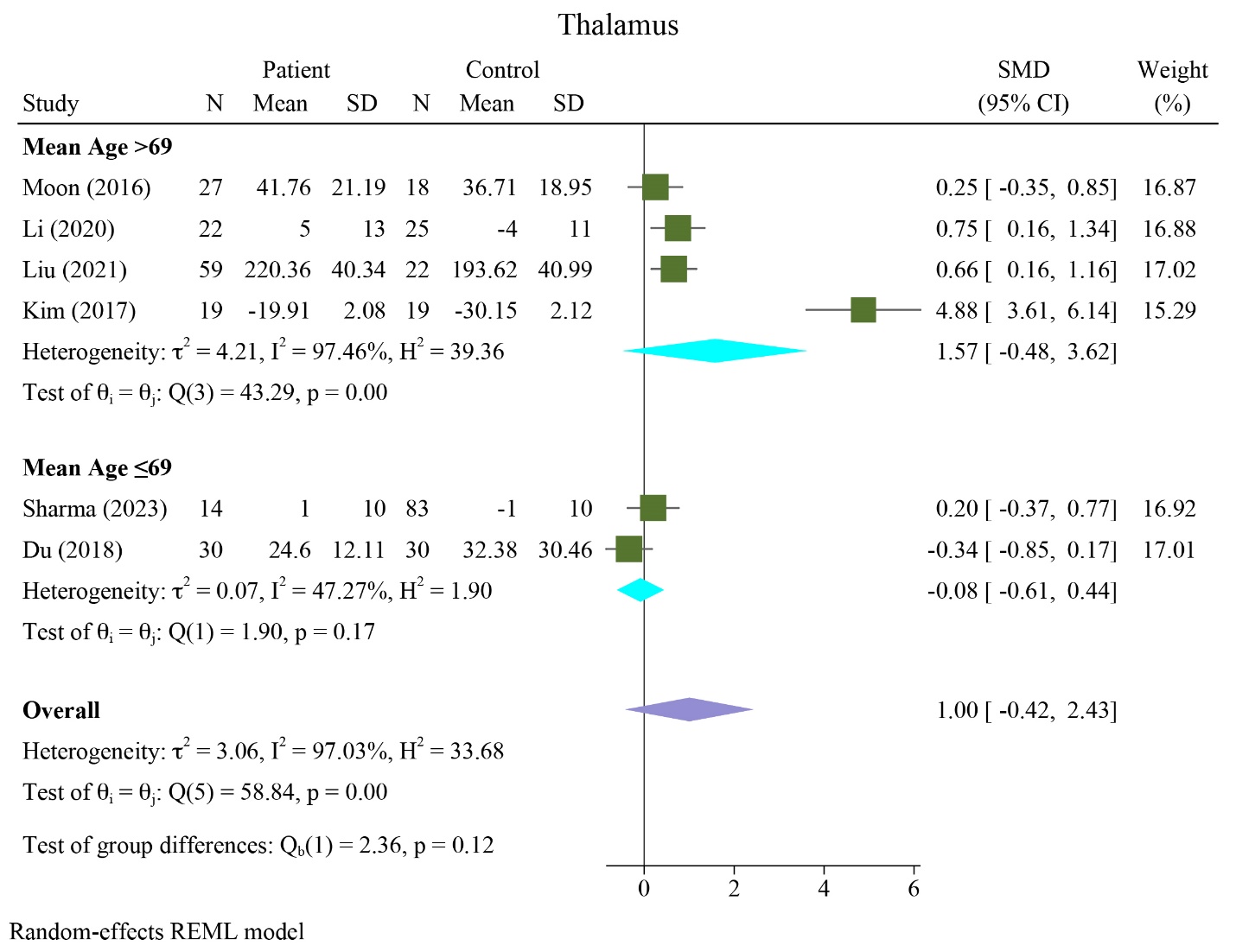


**FIGURE S11** Subgroup analysis results of QSM values based on ROB assessments in the caudate nucleus


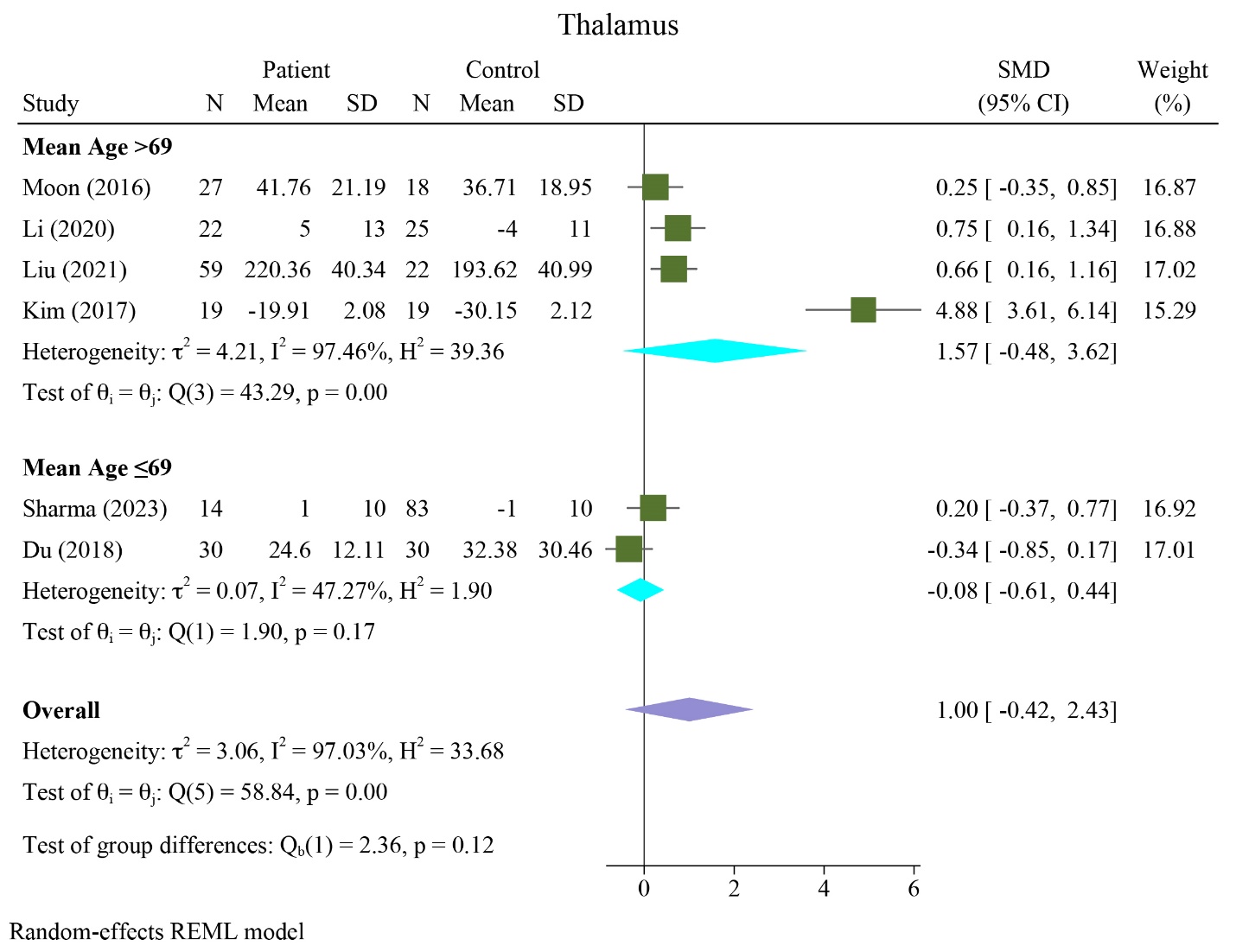


**FIGURE S12** Subgroup analysis results of QSM values based on age differences in the thalamus


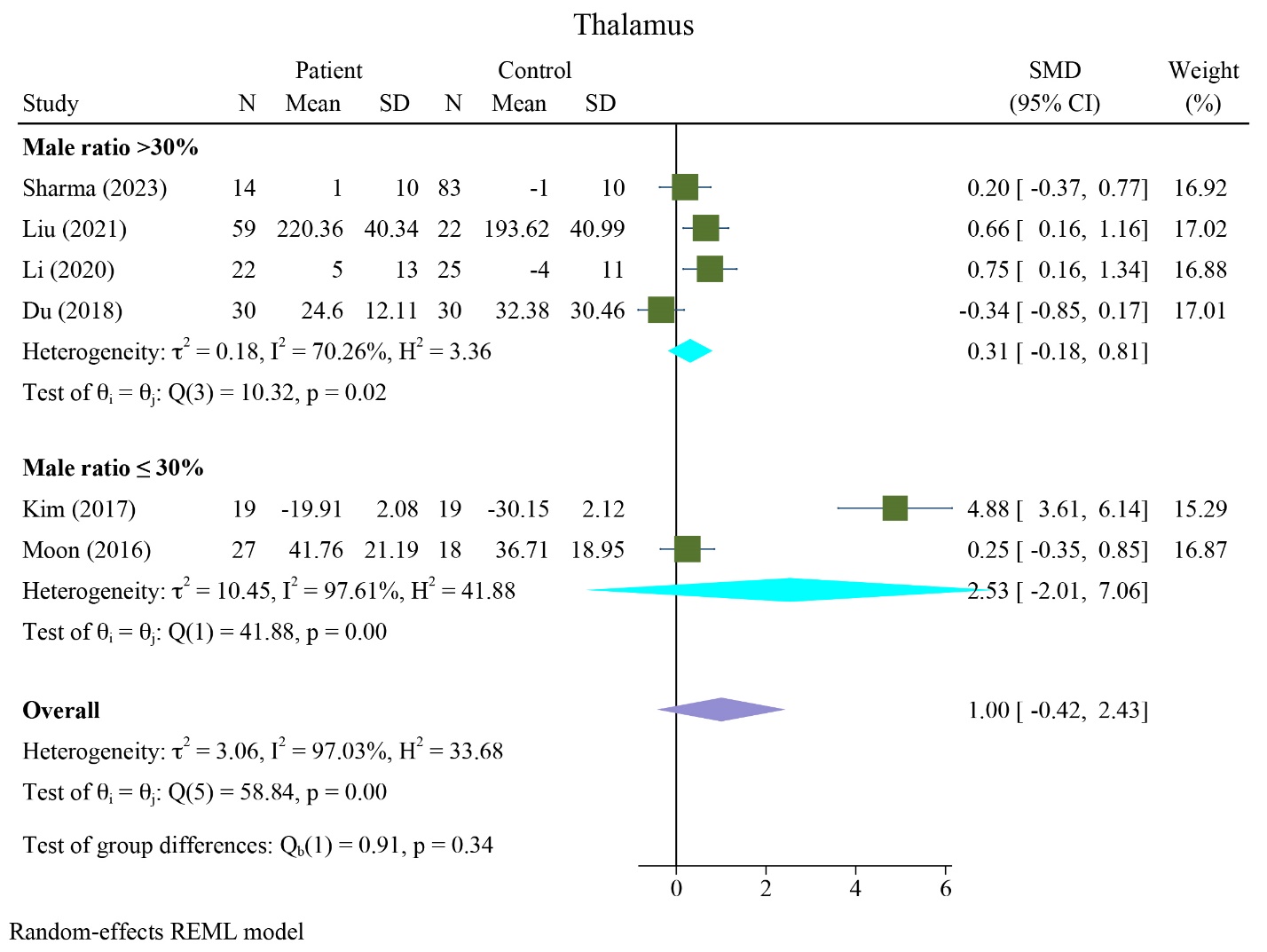


**FIGURE S13** Subgroup analysis results of QSM values based on sex differences in the thalamus


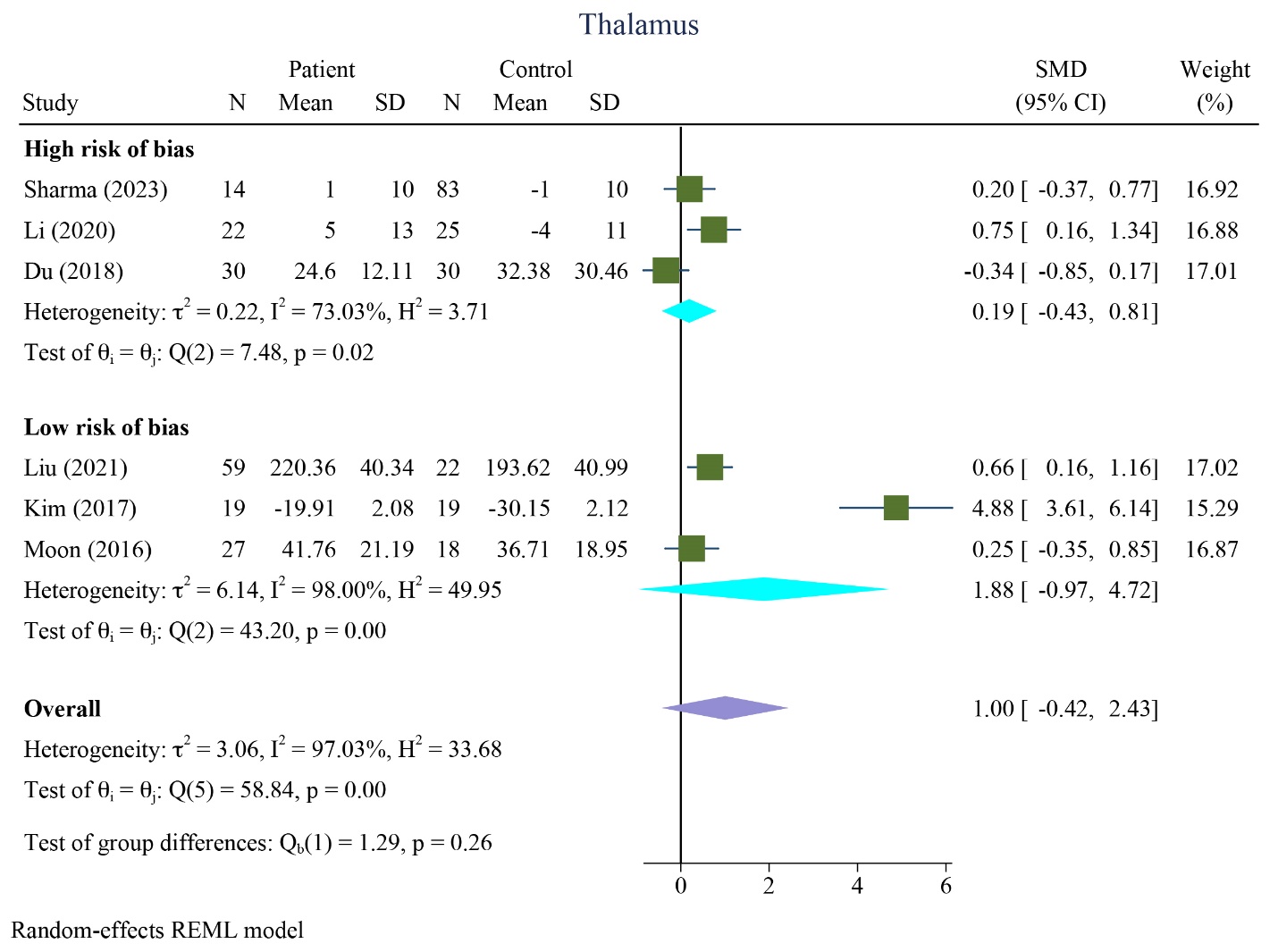


**FIGURE S14** Subgroup analysis results of QSM values based on ROB assessments in the thalamus


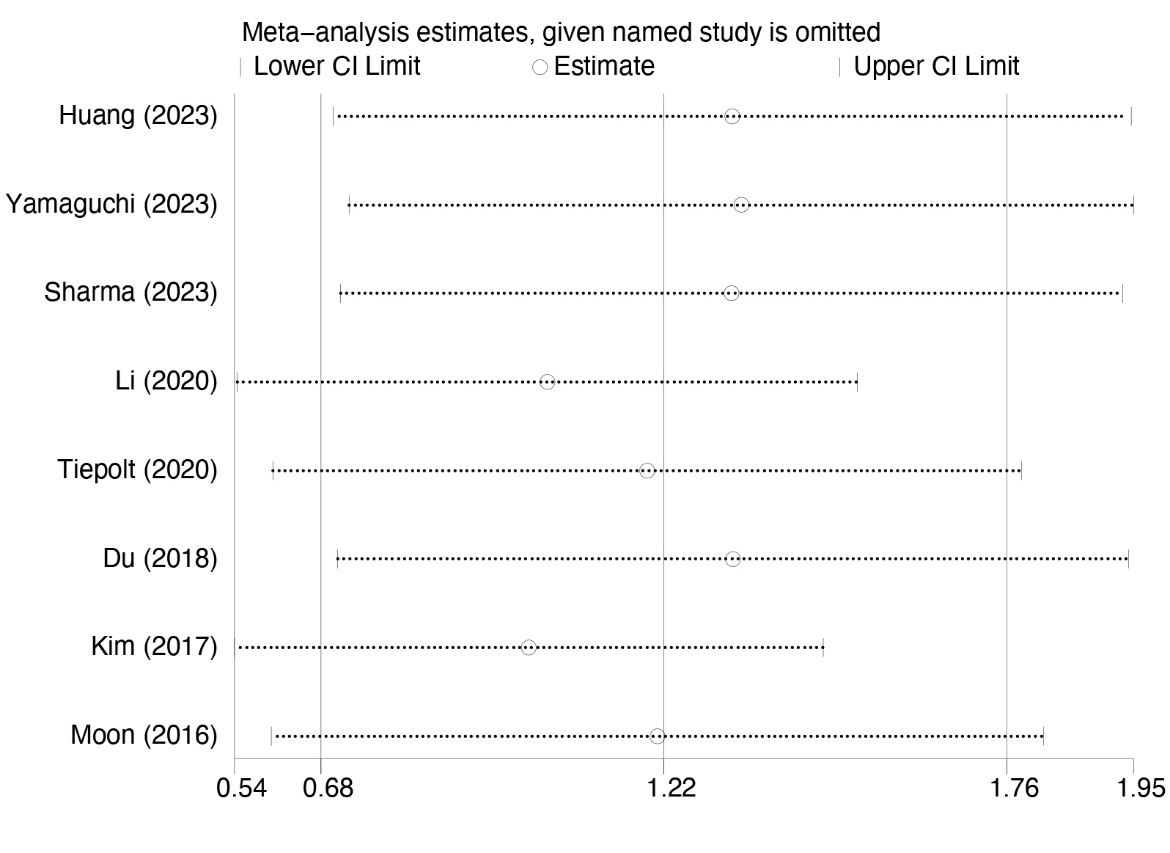


**FIGURE S15** Sensitivity analysis
